# pSB3, a member of the 2-micron family of plasmids, replicates and is efficiently partitioned in multiple budding yeast species

**DOI:** 10.1093/g3journal/jkag012

**Published:** 2026-02-18

**Authors:** Anastasiia Mereshchuk, Joyce S K Chew, Melanie J Dobson

**Affiliations:** Department of Biochemistry and Molecular Biology, Faculty of Medicine, Dalhousie University, 1459 Oxford Steet, Halifax, Nova Scotia, Canada B3H 4R2; Department of Biochemistry and Molecular Biology, Faculty of Medicine, Dalhousie University, 1459 Oxford Steet, Halifax, Nova Scotia, Canada B3H 4R2; Department of Biochemistry and Molecular Biology, Faculty of Medicine, Dalhousie University, 1459 Oxford Steet, Halifax, Nova Scotia, Canada B3H 4R2

**Keywords:** protein–DNA binding, chromosome segregation, plasmid inheritance, 2-micron plasmid, Rep1, Rep2, Flp recombinase, extrachromosomal elements, eukaryotic plasmids, partitioning proteins, *PAR* loci, chromatin, yeast vectors, *Lachancea*, *Saccharomyces*, *Torulaspora*, *Zygosaccharomyces*

## Abstract

The plasmids pSB3 of *Zygosaccharomyces rouxii* and the 2-micron circle of *Saccharomyces cerevisiae* belong to a small family of multi-copy yeast DNA plasmids. Members are similarly organized, with each encoding a conserved version of Flp, the recombinase required for copy number amplification. In contrast, proteins and loci required for partitioning seemed to differ. For the 2-micron circle, partitioning involves association of plasmid proteins Rep1 and Rep2 with each other and with the plasmid *STB* locus. Here, updated sequencing revealed all members encode a Rep1 protein more conserved than previously reported with pSB3 Rep1 the largest due to a long internal non-conserved region. pSB3 partitioning protein C, despite not resembling Rep2, was found to associate with pSB3 Rep1  *in vivo*, with amino-terminal domains of each sufficient for this interaction. Plasmid inheritance assays identified a region upstream of the *REP1* gene as the pSB3 partitioning locus (*PAR*) and showed pSB3 could replicate and partition in several budding yeast species including *Lachancea waltii*, *S. cerevisiae*, *Torulaspora delbrueckii*, and *Zygosaccharomyces bailii*. In one-hybrid protein–DNA interaction assays, pSB3 Rep1 and C were found to require each other for association with pSB3 *PAR* and the plasmid gene promoters and did not recognize 2-micron *STB*. Taken together, these results support the mechanism of partitioning and regulation of plasmid gene expression by partitioning proteins being conserved between pSB3 and 2-micron. Furthermore, molecular tools developed in this study provide the basis for developing new plasmid vectors based on pSB3 that can be effectively inherited in multiple budding yeast species.

## Introduction

**Fig. 1.**
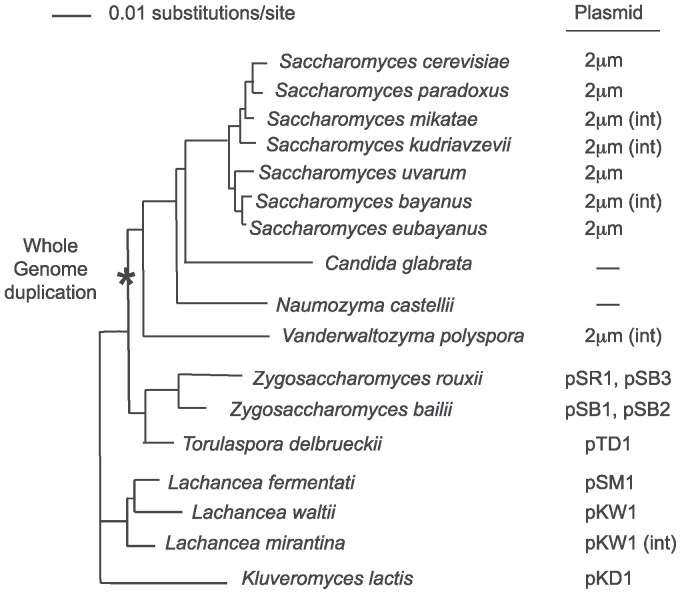
Occurrence of 2μm family plasmids in Saccharomycetaceae budding yeast species. The phylogenetic relationship of yeast species currently known to contain an autonomously replicating 2μm family plasmid or remnants of one integrated in the genome (int), or where no plasmid sequences have been identified (−), is shown. An asterisk indicates the whole genome duplication event in the yeast lineage. The figure was adapted from (Hedtke et al. 2006; Scannell et al. 2007; OhEigeartaigh et al. 2011; Pereira et al. 2011; Wolfe et al. 2015), with 2μm plasmid distribution reported by Frank and Wolfe (2009) and Strope et al. (2015).

pSB3 is one of a small number of circular double-stranded DNA plasmids to have been identified in several closely related budding yeast species (for review see, Utatsu et al. 1987; Volkert et al. 1989) (Fig. 1). The most intensively studied member of this family, the 2-micron circle (hereafter referred to as 2μm) is found at high copy number in the nucleus of most strains of the baker's yeast, *Saccharomyces cerevisiae* (Buchman et al. 1988; Strope et al. 2015). Plasmid presence confers no obvious benefit and minimal detriment to the host cells (Futcher and Cox 1983; Mead et al. 1986). These features have made 2μm the basis of many high-copy number vectors for biotechnological and molecular genetic applications (Mereshchuk et al. 2021).

**Fig. 2.**
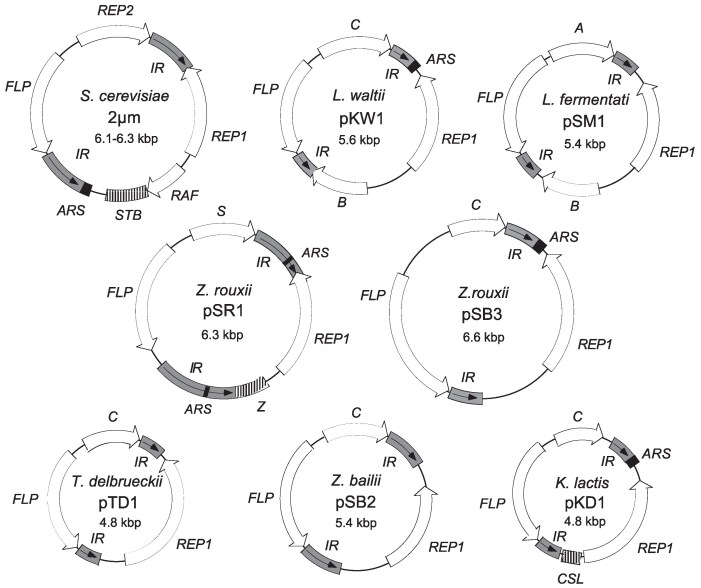
2μm family plasmids. The organization of currently known family members is shown in the B form with the size, and host species indicated. The *Z. bailii* pSB1 plasmid was not included as the sequence is unknown (Toh-e et al. 1984). Positions of protein-coding genes (white arrows), inverted repeats (*IR*; dark gray boxes with arrows showing orientation), *ARS* elements (*ARS*; black box), and experimentally determined partitioning loci, 2μm *STB* (Kikuchi 1983; Jayaram et al. 1985; Murray and Cesareni 1986), pSR1 Z (Araki et al. 1993), and the pKD1 *cis*-acting stability locus (*CSL*) (Bianchi et al. 1991), (striped boxes) are shown.

Members of this plasmid family do not share significant nucleotide sequence identity with 2μm or each other but all are of similar size and organization, with each having two unique regions separated by a pair of inverted repeats (*IR*s), and with some, like pSB3, having only three, rather than four open reading frames (ORFs) (Fig. 2) (Utatsu et al. 1987; Murray et al. 1988). Other members are pKD1 of *Kluyveromyces lactis* (Chen et al. 1986), pKW1 of *Lachancea waltii* (Chen et al. 1992), pSM1 of *Lachancea fermentati* (Utatsu et al. 1987), pSB1 and pSB2 of *Zygosaccharomyces bailii* (Toh-e et al. 1984), pSR1 of *Zygosaccharomyces rouxii* (Toh-e et al. 1982), and pTD1 of *Torulaspora delbrueckii* (Blaisonneau et al. 1997). Minor sequence variants of 2μm and other members have been identified in genome sequencing surveys and other studies (Xiao et al. 1991a , 1991b; Xiao and Rank 1993; Bolotin-Fukuhara et al. 2000; Frank and Wolfe 2009; Strope et al. 2015; Ortiz-Merino et al. 2017).

The 2μm family plasmids have been found to be stably maintained at high copy number in their respective hosts where they exist in equimolar amounts of two isomeric forms (A and B), products of recombination between sites in their IRs, catalyzed by the conserved site-specific recombinase Flp encoded by each (Toh-e et al. 1984; Araki et al. 1985; Toh-e and Utatsu 1985; Chen et al. 1986, 1992; Utatsu et al. 1987; Blaisonneau et al. 1997). For 2μm, this Flp-mediated recombination has been proposed to trigger copy number amplification by inverting one of the two bi-directional replication forks initiating from the plasmid's sole replication origin (*ARS*, autonomous replication sequence) during plasmid copying (Futcher 1986). This spools out a multimeric plasmid sequence until replication is terminated by a second recombination event that allows the replication forks to meet (Futcher 1986; Volkert and Broach 1986). The multimer can subsequently be converted to multiple monomers by Flp or host recombination with the net result being an increase in plasmid copy number. The similar organization of the 2μm family plasmids, with each having target sites in their IRs specific for the recognizable Flp protein they encode, supports Flp-mediated copy amplification being a conserved mechanism for all family members.

By contrast, the components responsible for partitioning of the 2μm family are much less conserved. Partitioning of roughly equal numbers of copies of the 2μm plasmid to each of the two products of host cell division requires the association of the plasmid Rep1 and Rep2 proteins with a repeated DNA sequence at the plasmid partitioning locus (*STB*) (Fig. 2) (Yang et al. 2004; Ghosh et al. 2006; McQuaid et al. 2019a). In the absence of either one of the Rep proteins or *STB*, plasmids fail to be equally partitioned and show a strong maternal segregation bias, with few daughter cells receiving plasmids when they bud from the mother cell (Murray and Szostak 1983; Jayaram et al. 1985). While other plasmids in this family are predicted to encode a protein with at least some limited sequence similarity to Rep1, none encode a recognizable Rep2 homolog, and none have a tandemly-repeated sequence at their partitioning locus. Even for 2μm, the mechanism that underlies equipartitioning remains to be established. Current evidence supports a “chromosome hitchhiking” model in which the Rep proteins complexed at *STB* enable plasmid attachment to segregating chromosomes although the nature of the attachment remains unclear (Sau et al. 2019; Ma et al. 2023; Girard et al. 2025).

In addition to a role in plasmid partitioning, the 2μm Rep proteins, also regulate plasmid gene expression, repressing transcription directed by two divergent promoter regions on the plasmid (Fig. 2) (Murray et al. 1987; Reynolds et al. 1987; Som et al. 1988; Veit and Fangman 1988; McQuaid et al. 2017). Repression of the *FLP* gene prevents plasmid number from exceeding the normal level, with increased Rep protein levels being the signal that prevents further increases in copy number. For 2μm, this Rep protein-mediated repression can be alleviated by Raf (Murray et al. 1987), the only other protein encoded by the plasmid, which competes with Rep2 for Rep1 association, disrupting the Rep1/Rep2 repressor complex, and potentially contributes to partitioning by interacting with and promoting Rep1 post-translational stability (McQuaid et al. 2017; Rizvi et al. 2017).

While the control of 2μm plasmid copy number has largely been elucidated (Futcher 1986; Volkert and Broach 1986; Som et al. 1988), the mechanism by which plasmid copies are equally partitioned at host cell division, and the role the plasmid partitioning proteins play in this process, are still not fully understood (for review, Rizvi et al. 2018). Here, we have investigated the partitioning system of a different member of the 2μm family of plasmids, that of the plasmid pSB3 of the osmophilic yeast *Z. rouxii* (Toh-e et al. 1984; Toh-e and Utatsu 1985). Resequencing and re-annotation of previously published sequences showed the Rep1 proteins encoded by pSB3 and those of other family members to be more similar than previously reported. The pSB3 partitioning proteins Rep1 and protein C were found to interact *in vivo* through domains similar to those that mediate 2μm Rep1 interaction with Rep2, and to associate with the pSB3 sequences that function in *cis* to increase plasmid inheritance in *Z. rouxii,* akin to Rep protein/*STB*-mediated 2μm plasmid partitioning. A pSB3-based yeast-bacterial shuttle plasmid was stably maintained at high copy number in *S. cerevisiae,* even when the pSB3 *FLP* gene or a non-conserved internal region of the *REP1* gene were deleted and was efficiently partitioned in multiple budding yeast species indicating pSB3 might prove a versatile basis for high copy number vectors. This study is the first to analyze protein and DNA associations for the partitioning proteins of another member of the 2μm plasmid family, providing insight into the components that have diverged and those that have been constrained during plasmid evolution from their last common ancestor.

## Materials and methods

### Strains and media

Yeast strains used is this study are listed in Supplementary Data Table 1. Strains lacking a 2μm family plasmid are designated as *[cir^0^]*. Yeast cells were cultured at 28°C in YPAD (1% yeast extract, 2% Bacto Peptone, 0.003% adenine, 2% glucose), in synthetic complete (SC) (0.67% Difco yeast nitrogen base without amino acids, 2% glucose, 1% Difco casamino acids, 0.003% adenine, 0.002% uracil, 0.002% tryptophan), or in synthetic defined (SD) medium (0.67% Difco yeast nitrogen base without amino acids, 2% glucose, 0.003% adenine, 0.002% uracil, and all required amino acids (Burke et al. 2000). To select for the presence of plasmids with specific nutritional marker genes, SC or SD medium lacking specific bases or amino acids was used. To select for the presence of *kanMX*-tagged plasmids, YPAD supplemented with 200 mg/L geneticin (G418, Sigma) was used. Solid media were prepared by addition of 2% agar. Yeast cells were transformed using the LiAc/SS-DNA/PEG method (Gietz et al. 1995) with the following adaptions for non-*S. cerevisiae* yeast (Branduardi et al. 2004). Transformed cells were allowed to recover in YPAD containing 1 M sorbitol (YPAD/S), or for *Z. bailii* in YPAF/S (1% yeast extract, 2% Bacto Peptone, 0.003% adenine, 2% fructose, 1 M sorbitol), for 16 h at 28 °C before plating on YPAD/S or YPAF/S solid medium, respectively, containing 200 mg/L G418.

### Plasmids

Plasmids pSB3 and pSR1 were isolated from *Z. rouxii* yeast cells containing both plasmids (ATCC strain 56076). Genomic DNA was extracted from the yeast, digested with XhoI, and resolved by agarose gel electrophoresis. Fragments of ∼7 and ∼6 kbp, corresponding to the full-length plasmids, were extracted from gel slices and cloned in SalI-digested pTZ18R (Pharmacia), giving plasmids pTZ-pSB3 and pTZ-pSR1, respectively. Plasmid identities were confirmed by sequencing.

Other plasmids and oligonucleotides used as primers for polymerase chain reactions (PCR) in this study are listed in Supplementary Data Tables 2 and 3, respectively. All sequences generated by PCR were confirmed by sequencing.

### DNA and protein sequence analysis

Sequences of 2μm family plasmids and conceptual translations of their ORFs were obtained from https://www.ncbi.nlm.nih.gov and had the following Genbank accession numbers: the 2μm variant Scp1 found in most laboratory strains of *S. cerevisiae* (J01347.1), *T. delbrueckii* pTD1 (Y11042), *Z. rouxii* pSR1 (X02398.1), *Zygosaccharomyces parabailii* pSB2 (CP019507.2), *L. fermentati* pSM1 (M18275), *L. waltii* pKW1 (X56553.1), and *K. lactis* pKD1 (X03961.1). The *Z. rouxii* pSB3 plasmid and the ORFs encoding the pKW1 and pSR1 partitioning proteins were re-sequenced here, the former from overlapping reads of cloned plasmid DNA, and the latter from PCR products amplified from yeast containing the plasmids. The updated sequences of the pSB3 plasmid (PV135004) and pKW1 *REP1* ORF (PV135003) replace prior versions (X02608 and X56553). A BLASTP search with the corrected pSB3 Rep1 protein sequence (XPQ57843) identified one other pSB3 Rep1 protein (ZYGR_0R01040), encoded in the genome of an allopolyploid *Z. rouxii* strain NBRC 110957 (GAV4986.1). The previously reported pSB2 sequence (Utatsu et al. 1987) had errors that truncated the *REP1* ORF. A corrected sequence was reported (CP019507.2) (Ortiz-Merino et al. 2017) but the *REP1* ORF was not identified. A pSB2 variant, pZB1 (PX275371), was identified in a natural isolate of *Z. bailii,* and was 90.6% identical to pSB2 (Dobson, unpublished results). The pKD1 sequence has been reconfirmed in whole genome surveys (AL427257, AL427843, AL429084) (Bolotin-Fukuhara et al. 2000). pSM1 has not been re-sequenced but an additional C added to a run of C nucleotides at position 2,804 in the previously reported pSM1 plasmid sequence (M18275) gives an ORF that encodes a 394-amino acid Rep1 protein rather than the reported 200-residue amino-terminally-truncated protein. Here, the longer version was considered correct. The plasmid maps shown in Fig. 2 are based on the corrected sequences for pSB2, pSB3, pSM1, and pKW1. Accession numbers and details of yeast plasmid proteins analyzed in this study are listed in Supplementary Data Tables 4 and 5.

DNA and protein sequences were analyzed using DNA Strider 3.0 (Marck 1988). Predicted protein sequences were aligned using Clustal Omega with default settings (Madeira et al. 2024) and shaded using Boxshade 3.21 (accessed through www.ebi.ac.uk/). Conserved motifs were identified using GLAM2 (Frith et al. 2008). Predicted three-dimensional structures of proteins were obtained from the AlphaFold Protein Structure Database (AFDB) (alphafold.ebi.ac.uk) or generated using AlphaFold (alphafoldserver) (Jumper et al. 2021; Fleming et al. 2025). Domain predictions were obtained from The Encyclopedia of Domains (Lau et al. 2024) accessed through AFDB. Similarities between protein structures were sought using MMSeqs2 (Steinegger and Soding 2017) and Foldseek (van Kempen et al. 2024) accessed through AFDB or the Foldseek server (search.foldseek).

### pSB3-based plasmids

**Fig. 3.**
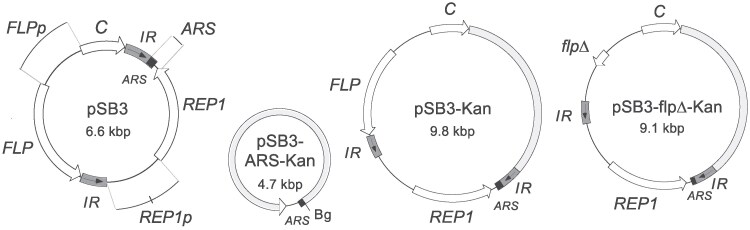
pSB3 and pSB3-based yeast-*E.coli* shuttle plasmids. pSB3 *REP1* and *FLP* gene promoter regions (*REP1p* and *FLPp*) and the *ARS*-containing fragment used in functional studies are indicated outside the pSB3 map. In shuttle plasmids, bacterial vector sequence (thick pale grey line) includes an *E.coli* origin of replication and genes conferring ampicillin-resistance in bacteria and G418-resistance in yeast. The position of the BglII site (Bg) used for cloning in pSB3-ARS-Kan is shown.

A yeast-*E. coli* shuttle plasmid based on pSB3 (pSB3-Kan) was created by inserting pUC18 vector and *kanMX* gene sequences between two MluI sites downstream of the *C* gene in pSB3 (see Supplementary Data for details) (Genbank PV135005) (Fig. 3). Other than the 9-bp MluI fragment replaced by vector insertion, pSB3-Kan contains all other pSB3 plasmid sequences, and no protein coding regions are disrupted. To create a version of pSB3-Kan lacking a functional *FLP* gene (pSB3-flpΔ-Kan), pKan-pSB3 was digested with AgeI/StuI, the overhangs filled in, and the plasmid circularized by ligation. To create versions of pSB3-Kan with a *REP1* ORF lacking codons 255 to 377, either with an intact *FLP* ORF (pSB3-REP1Δ123-Kan), or with the *FLP* ORF deleted (pSB3-flpΔ-REP1Δ123-Kan), a 1.0 kbp SstII/BglII fragment in pSB3-Kan and pSB3-flpΔ-Kan, respectively, was replaced with a 0.7 kbp SstII/BglII fragment isolated from a PCR-generated, internally deleted, version of the *REP1* gene.

To create a *kanMX*-tagged yeast-*E. coli* shuttle vector that could replicate but not partition in yeast, a 383-bp stretch of pBS3 consisting of the region previously shown to be sufficient for autonomous replication in *S. cerevisiae* (Toh-e and Utatsu 1985), but also including the adjacent *REP1* gene transcriptional termination sequence, was amplified with flanking SstI and EcoRI sites and a BgllII site and cloned in SstI/EcoRI-digested pTZ18R, creating pSB3-ARS-Kan (Fig. 3). The reported HindIII-BanII *ARS*-containing fragment from pSB3 was initially tried but yeast transformation efficiency was significantly improved by inclusion of the transcriptional termination region (A. Mereshchuk, data not shown).

### Plasmids for expression of yeast plasmid proteins as fusion proteins

Plasmids that in yeast constitutively express the *S. cerevisiae* 2µm Rep1, Rep2, and Raf proteins fused to the transcriptional activation domain of Gal4 (Gal4_AD_; pGADREP1, pGADREP2, and pGADRAF) or fused to the DNA-binding domain of the bacterial repressor protein LexA (amino acids 1 to 87) (LexA_BD_) (pSHREP1, pSHREP2, and pSHRAF), have been previously described (Sengupta et al. 2001; McQuaid et al. 2017). Here plasmids to express pSR1 Rep1 and S proteins, the pSB3 Rep1 and C proteins, and truncated versions of these, fused to Gal4_AD_ or to LexA_BD_ (hereafter referred to as AD and BD, respectively), were similarly created by PCR amplification of the coding regions for each flanked by restriction sites that allowed them to be cloned in-frame with the AD or BD ORFs in pGAD424 and pSH2-1 vectors, respectively (for details, see Supplementary Data Tables 2 and 3).

### Reporter plasmids for one hybrid assays

Plasmids encoding a DNA sequence of interest upstream of a *lacZ* reporter gene were used to create one-hybrid yeast reporter strains to test partitioning protein recognition of partitioning DNA sequences *in vivo*. The pSR1 *Z* partitioning locus (Araki et al. 1993), the pSB3 *REP1* gene promoter region extending up to the adjacent inverted repeat (IR) sequence, and the pSB3 *FLP* and C gene divergent promoter region (*FLPp*) were amplified from the native plasmids flanked by BglII and BamHI restriction sites. The resulting fragments were cloned in the *URA3* gene-tagged one-hybrid vector pJL638 (Li and Herskowitz 1993) at a BglII site upstream of the *lacZ* reporter gene, creating the pJL638 plasmid series (for details, see Supplementary Data Tables 2 and 3). To integrate the reporter genes into the yeast genome, pJL638 plasmids were linearized with StuI, which cuts within the *URA3* gene in pJL638 and used to transform *S. cerevisiae* strain JP49/6b (lacks 2μm; *ura3-1*) to uracil prototrophy.

### Plasmid inheritance assays

Inheritance of *kanMX*-tagged plasmids was monitored as previously described (Pinder et al. 2013). G418-resistant yeast transformants were cultured for 16 to 24 h (∼10 generations) in selective medium (YPAD + G418). The mitotic stability (defined as the percentage of cells containing the plasmid in a population of cells grown in medium selective for the plasmid) was determined from the ratio of viable colonies obtained by plating on medium selective for the plasmid (YPAD + G418) vs non-selective (YPAD) medium. The plasmid loss rate during culture in non-selective medium was determined as previously described (Murray and Cesareni 1986). For all inheritance assays results represent the average with standard deviation from analyzing a minimum of four yeast transformants for each unless otherwise indicated. Statistical significance was assessed using a two-tailed Student's *t*-test.

### Two-hybrid assays

Protein–protein association was assayed in a [*cir^0^*] *S. cerevisiae* yeast strain (CTMD/3a) with 8 copies of the LexA operator sequence in a basal promoter region upstream of a *lacZ* reporter gene integrated in the genome at the *URA3* locus (Hanes and Brent 1989). The reporter strain was co-transformed with two plasmids expressing the proteins to be assessed for interaction, one (pGAD-based) expressing an AD fusion protein and the other (pSH-based) expressing a BD fusion protein. Activation of the *lacZ* gene, an indication of interaction of the two hybrid proteins, was monitored using a β-galactosidase filter assay.

### One-hybrid assays

Protein–DNA interactions *in vivo* were assessed by expressing a protein of interest fused to AD in a [*cir^0^*] *S. cerevisiae* yeast reporter strain (JP49/6b) with the target DNA sequence integrated in the genome upstream of the *lacZ* reporter gene. For assays where the 2μm or pSB3 Rep1 protein was co-expressed in the cells with AD or an AD fusion, these were expressed constitutively as BD fusions from a second pSH-based plasmid. Expression of the reporter was monitored as in the two-hybrid assay.

## Results

### The Rep1 proteins encoded by 2μm family plasmids are more similar than previously reported

**Fig. 4.**
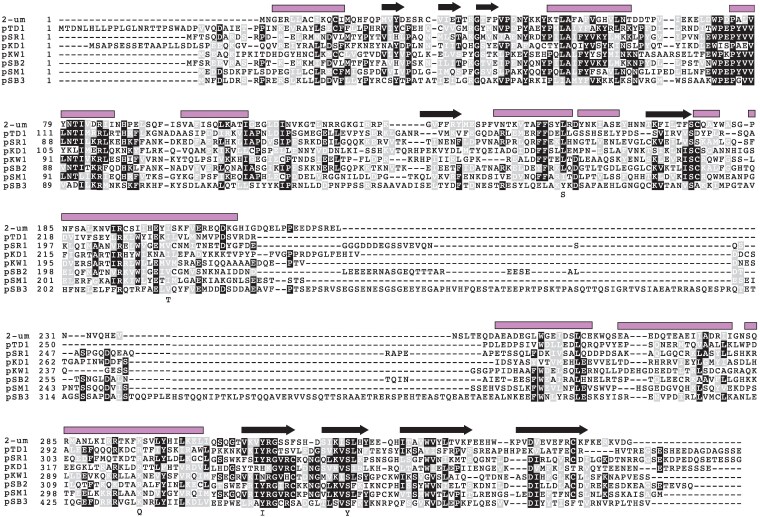
Clustal alignment of Rep1 protein sequences encoded by representative members of the 2μm plasmid family. Plasmid names are shown at left. The 2μm sequence is that of the Scp1 variant (P03871). Residues conserved at a given position in at least 50% of sequences are indicated by shading, black boxes for identical amino acids and grey for similar (Boxshade). Positions of alpha helices (purple bars) and beta strands (black arrows) predicted by AlphaFold in 2μm Rep1 are shown above the alignment. Amino acid substitutions in 2μm Rep1 that led to loss of *STB* association while not abolishing interaction with Rep2 are shown beneath (Yang et al. 2004; Pinder et al. 2013).

Sequences of 2μm family plasmids determined in early studies predicted some encoded a partitioning protein with only limited similarity to 2μm Rep1 (Utatsu et al. 1987; Volkert et al. 1989). For pSB3, the Rep1-related protein seemed to lack the 2μm Rep1 amino-terminal region required for Rep2 interaction (Sengupta et al. 2001), while pKW1 Rep1 seemed to be missing carboxy-terminal sequence motifs found in other Rep1-related proteins. Here re-sequencing revealed pSB3 to have a much longer *REP1* ORF than previously reported, encoding a 517-residue, rather than a 322-residue protein. A missing nucleotide in the earlier sequence had introduced a frameshift that prematurely terminated the predicted *REP1* ORF. Alignment of conceptual translations of the corrected pSB3 and pKW1 *REP1* ORFs with those of other members of the 2μm plasmid family shows the Rep1 proteins to be more conserved than previously reported (Fig. 4). Sequence motifs shared between the different Rep1 proteins were identified in amino-terminal (residues 30 to 63 and 69 to 92 for 2μm Rep1) and carboxy-terminal regions (residues 300 to 347 for 2μm Rep1) (Supplementary Data Fig. 1). The most significant variation between the proteins was observed in a central region between the more conserved amino- and carboxy-terminal regions, with pSB3 Rep1 having the longest unmatched internal stretch, and *T. delbrueckii* pTD1 Rep1 the shortest. Secondary structure analysis predicted this non-conserved internal region of pSB3 Rep1 to be largely unstructured while regions displaying higher similarity between the Rep1 proteins corresponded to sequences predicted to form alpha-helices or beta-strands (Fig. 4).

### Protein modeling predicts Rep1 domain structure is conserved

**Fig. 5.**
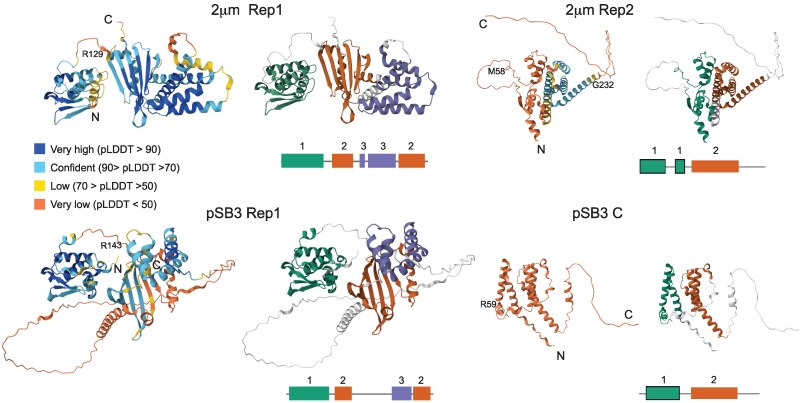
Predicted structures of the 2μm and pSB3 Rep1 proteins. AlphaFold models of *S. cerevisiae* 2μm Rep1 (P03871) and Rep2 (P03872) (*top*), and *Z. rouxii* pSB3 integrated *REP1* variant ZYGR_0R01040 (A0A1Q3A2B5) and protein C (A0A286RWR0) (*bottom*), are shown (left) visualized in colors that indicate the per-residue model confidence score (pLDDT) between 0 and 100. Predicted domains are indicated by color (*right*) with position of sequences comprising each domain shown schematically beneath. Note, AlphaFold does not reliably predict the position of the domains relative to one another for these proteins. The amino (N) and carboxy (C) termini and relevant amino acid positions are indicated.

Three-dimensional structures have not been determined experimentally for any of the 2μm family plasmid partitioning proteins, but structures of some have been predicted by AlphaFold protein modeling (Jumper et al. 2021; Varadi et al. 2024; Fleming et al. 2025) (Fig. 5 and Supplementary Data Figs. 2 and 3) (*Note: some Rep1 protein structures in the AlphaFold Protein Database were not considered here as they were based on plasmid sequences that have since been found to be incorrect—*see Materials and Methods and Supplementary Data Tables 4 and 5). Comparison of the structures predicted for Rep1 encoded by the 2μm variant Scp1 vs that encoded by a pSB3 variant GAV4986, shows a similar organization (Fig. 5). Both are predicted to have an independently-folded amino-terminal domain, consistent with experimental data for 2μm Rep1, showing the first 129 amino acids is sufficient for Rep2 interaction (Sengupta et al. 2001). The remaining portion of both proteins is predicted to be organized into two further domains, one characterized by a beta-sheet formed by alignment of six beta-strands with one, or possibly two, alpha-helices participating, and the other consisting of four alpha-helices encoded by the stretch between the two beta strand-encoding regions. The long internal non-conserved sequence in this variant of pSB3 Rep1 was not predicted to be part of any of the domains although it should be noted that the confidence level for this part of the structure is very low. AlphaFold modeling also predicted similar three-dimensional organization for Rep1 proteins encoded by the other 2μm family plasmids, with some having two, rather than three domains (Supplementary Data Fig. 2). Foldseek searches also supported all currently known Rep1 proteins as sharing significant structural similarity (Supplementary Data Tables 4 and 5).

### Comparison of Rep2-equivalent proteins encoded by 2μm family plasmids

In contrast to the similarity between Rep1 proteins encoded by different members of this plasmid family, none of the Rep1 partitioning partner proteins share significant sequence similarity with *S. cerevisiae* 2μm Rep2 (McQuaid et al. 2017). Similarity is seen between the *Lachancea* pSM1 A and pKW1 C proteins, and between the *Zygosaccharomyces* pSB2 C and pSR1 S proteins which, for each pair, likely reflects the closer phylogenetic relationship between their respective yeast hosts (Fig. 1). The pKD1, pTD1 and pSB3 C proteins do not share sequence similarity. Foldseek searches also did not identify similarity between the structures predicted for them, or between them and any other proteins in the AlphaFold Protein Databases, although the confidence level for C protein models was very low (Fig. 5, Supplementary Data Fig. 3 and Supplementary Data Table 4).

Foldseek searches with pSM1 A did identify pKW1 C and 2μm Rep2 as sharing structural similarity although the reciprocal search with Rep2 identified no significant matches (Supplementary Data Table 4). One feature that was shared by the structures predicted for Rep2 and those of the other Rep2-equivalent proteins (with the exception of pKD1 C), was organization into two domains (Fig. 5 and Supplementary Data Fig. 3), with most predicted to have a long, primarily unstructured, C terminal tail. A similar two-domain organization was also predicted for the B proteins encoded by the *RAF*-positioned ORF in plasmids pSM1 and pKW1 and for the 2μm Raf protein, which like Rep2, heterodimerizes with Rep1 through an amino-terminal domain (McQuaid et al. 2017) (Supplementary Data Fig. 3). However, Foldseek searches did not identify significant similarity between the predicted structures of Raf and either of these B proteins or with any other proteins in the databases (Supplementary Data Table 4). Taken together the results of modeling and sequence analysis of the 2μm family plasmid partitioning proteins supports conservation of Rep1 protein structure and suggests the second partitioning protein for most of the plasmids has diverged from the ancestral version to the extent that similarity can no longer be detected although it is formally possible that some plasmid lineages had a different gene at that position.

### The pSB3 plasmid is efficiently partitioned in *S. cerevisiae* even when the *FLP* gene is deleted

Studies of the partitioning proteins of 2μm family plasmids, other than those of the 2μm plasmid, have largely been limited to identifying the genes encoding them. Investigating those of the *Z. rouxii* pSB3 plasmid was of particular interest given the large internal non-conserved region in the pSB3 Rep1 protein and the lack of similarity between the pSB3 C protein and 2μm Rep2. pSB3 has previously been shown to replicate in *S. cerevisiae,* with the region required for this autonomous replication localized to a 168-bp region adjacent to one of the IRs (Toh-e et al. 1984; Toh-e and Utatsu 1985) (Fig. 3). The pSB3 Flp recombinase was also functional in *S. cerevisiae* (Toh-e et al. 1984). Unfortunately, due to errors in the published sequence, plasmids created to test pSB3 partitioning function at that time had vector or marker gene sequences inadvertently inserted either within the C protein ORF or at a site which separated the *REP1* ORF from its promoter. Although the test plasmids partitioned in *Z. rouxii* cells which contained native pSB3 plasmid to supply the Rep1 and C partitioning proteins *in trans*, none were stably maintained in *S. cerevisiae* (Toh-e and Utatsu 1985). This led to the conclusion that the pSB3 partitioning mechanism was not functional in this heterologous yeast host. Resequencing of pSB3 suggested instead that test plasmid instability might have resulted from perturbed expression of the partitioning protein-encoding genes.

**Fig. 6.**
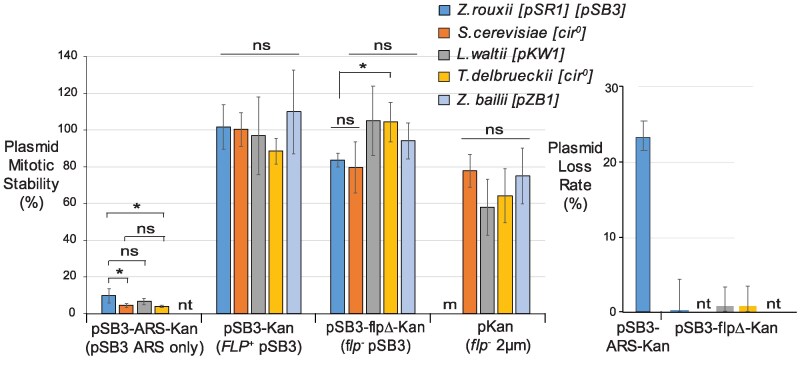
Inheritance of *kanMX*-tagged 2μm and pSB3 plasmids in different budding yeast species. Transformed yeast were cultured in medium selective for plasmid presence (YPAD + G418) and the percentage of cells resistant to G418 (an indication of plasmid presence) (*left*), and the percentage of plasmid-free cells arising per generation during culture in non-selective medium (YPAD) (*right*), were determined by plating assays. Results represent the average (±StDev) from analyzing 4 independent transformants for each plasmid (ns = not significant; * *P* < 0.05, nt = not tested; m = microcolonies obtained but failed to proliferate).

To determine whether pSB3 could be efficiently maintained in *S. cerevisiae*, pSB3 was tagged with a dominant drug-resistance marker gene (*kanMX*), creating plasmid pSB3-Kan (Fig. 3). *E. coli* vector sequences and the *kanMX* gene were inserted downstream of the transcriptional termination sequence for the *C* ORF and adjacent to one copy of the *IR* to limit the impact they might have on expression of the pSB3 genes or chromatin organization at the partitioning locus. As a control, maintenance of pSB3-Kan was compared to that of a *kanMX*-tagged 2µm plasmid (pKan) (Pinder et al. 2013). pKan consists of a complete copy of 2µm but has a disrupted *FLP* gene, making inheritance of the plasmid solely dependent on the 2µm partitioning system (Pinder et al. 2013). An amplification-defective version of pSB3-Kan (pSB3-flpΔ-Kan) was similarly created here by deleting an internal portion of the *FLP* coding region (Fig. 3). As a further control, we generated a *kanMX*-tagged plasmid (pSB3-ARS-Kan) containing only the region of pSB3 previously shown to be sufficient for autonomous replication in *Z. rouxii* and in *S. cerevisiae* (Toh-e and Utatsu 1985) and which would be expected to lack *cis*-acting sequences targeted by the pSB3 partitioning proteins. All plasmids were then assessed for inheritance in *Z. rouxii* and *S. cerevisiae* (Fig. 6).

pSB3-ARS-Kan which, as expected for a plasmid able to replicate but lacking an active partitioning mechanism, was able to efficiently transform both yeast species to G418-resistance but was present in only a low percentage of either cell population, despite cells being cultured in medium selective for plasmid presence. In contrast, the amplification-competent pSB3-Kan plasmid displayed high mitotic stability in both *Z. rouxii* and *S. cerevisiae* with virtually all cells capable of forming colonies on medium containing G418. The pSB3-flpΔ-Kan plasmid also showed high mitotic stability in both species, although slightly less than the amplification-competent pSB3-Kan. Gel electrophoretic analysis showed pSB3-flpΔ-Kan to be at high copy number in the *S. cerevisiae* transformants, with the copy number of pSB3-Kan higher still, consistent with the pSB3 amplification mechanism functioning in these cells (Supplementary Data Fig. 4) (Toh-e et al. 1984). Flp-mediated correction of even rare unequal partitioning events would be expected to increase the average plasmid copy number in the cell population. Although the mitotic stability of pSB3-flpΔ-Kan in both species was less than that of pSB3-Kan, the level was similar to that of the amplification-defective 2µm-based pKan plasmid in *S. cerevisiae*, indicating that the pSB3 partitioning mechanism is as efficient as that of the native 2μm plasmid in this yeast. Inheritance of the 2µm-based pKan plasmid in *Z. rouxii* could not be assessed as transformants were only able to form microcolonies and failed to proliferate when sub-cultured, consistent with prior reports that 2μm is unable to replicate efficiently in *Z. rouxii* (Araki et al. 1985).

Unexpectedly, when maintenance of pSB3-Kan and pSB3-flpΔ-Kan was monitored in plating assays, the number of colonies formed on solid medium containing G418 sometimes slightly exceeded that observed when the same number of cells was plated on medium lacking this translational-inhibitor (Fig. 6). This could be due to random variation in plating or might suggest a small fraction of the cells containing plasmid were failing to proliferate when the drug was removed. A possible explanation might be that the plasmid copy number in those cells was higher-than-average, a level that might be tolerated when translation was less efficient, but sufficient to be toxic when translational efficiency was suddenly increased by withdrawal of the drug. For either pSB3-Kan or pSB3-flpΔ-Kan, a higher copy number could arise in some cells from less-than-equal partitioning events and might represent a selective advantage as long as G418 was present. An elevated copy number of 2μm has been shown to block proliferation of *S. cerevisiae* cells (Dobson et al. 2005) and, even in the absence of plasmid, combined over-expression of Rep1 and Rep2 is sufficient to block progression through the G2/M phase of the cell cycle (Reynolds et al. 1987). Over-expression of pSB3 partitioning proteins in cells with very high plasmid copy number might produce a similar response. Accurate quantification of the pSB3-based plasmid copy number in these transformants is needed to assess this possibility.

### The pSB3 plasmid replicates and is efficiently partitioned in other budding yeast species

The efficient maintenance of the pSB3 plasmid in *S. cerevisiae* prompted us to assess inheritance in other budding yeast species. *L. waltii*, *T. delbrueckii*, and *Z. bailii*, were all found to maintain pSB3-Kan, whether amplification-competent, or with the *FLP* gene deleted as effectively as *Z. rouxii* (Fig. 6). This contrasted with the Flp-defective *kanMX*-tagged 2μm plasmid, pKan which, while capable of producing viable transformants in these three species, displayed lower mitotic stability than pSB3-flpΔ-Kan in the former two (*P* < 0.01). To determine the effectiveness of pSB3 partitioning in *L. waltii* and *T. delbrueckii*, the rate of loss of pSB3-flpΔ-Kan from transformants cultured in the absence of selection for plasmid presence was determined (Fig. 6). The amplification-defective pSB3-flpΔ-Kan was maintained as stably in both species as in the native *Z. rouxii* host. The loss rate (less than 5% of the cell population becoming plasmid-free per cell division) was comparable to that reported for the amplification-defective 2μm-based pKan plasmid from *S. cerevisiae* transformants cultured under the same conditions (Pinder 2011). Overall, the data show that pSB3 can be efficiently maintained in multiple budding yeast species and indicate that any host factors required for replication and partitioning of this plasmid are conserved between these species.

### The pSB3 Rep1 and C partitioning proteins interact in *S. cerevisiae*

Based on the mitotic stability of pSB3-flpΔ-Kan in *S. cerevisiae*, the pSB3 Rep1 and C proteins must be capable of fulfilling their partitioning function in this heterologous yeast host (Fig. 6). To investigate this further, the pSB3 proteins were assessed for self-association and for interaction with one another *in vivo* using a two-hybrid protein assay. For these, each was fused either to the transcriptional activation domain of the yeast Gal4 protein (AD) or to the DNA-binding domain of the bacterial repressor protein LexA (BD) and expressed in *S. cerevisiae* cells lacking native 2μm and having a *lacZ* reporter gene with LexA binding sites upstream. Activation of reporter gene expression above background levels indicates recruitment of AD to the promoter-bound BD due to interaction between the test proteins fused to each. As controls, interactions of the 2μm Rep1, Rep2 and Raf proteins were assessed in the same assay. The partitioning proteins encoded by the *Z. rouxii* plasmid pSR1 (Rep1 and protein S) were also tested as, like pSB3, pSR1 has been reported to be stably maintained in *S. cerevisiae* cells although the encoded proteins have not been investigated (Araki et al. 1985; Jearnpipatkul et al. 1987a; Araki and Oshima 1989).

**Fig. 7.**
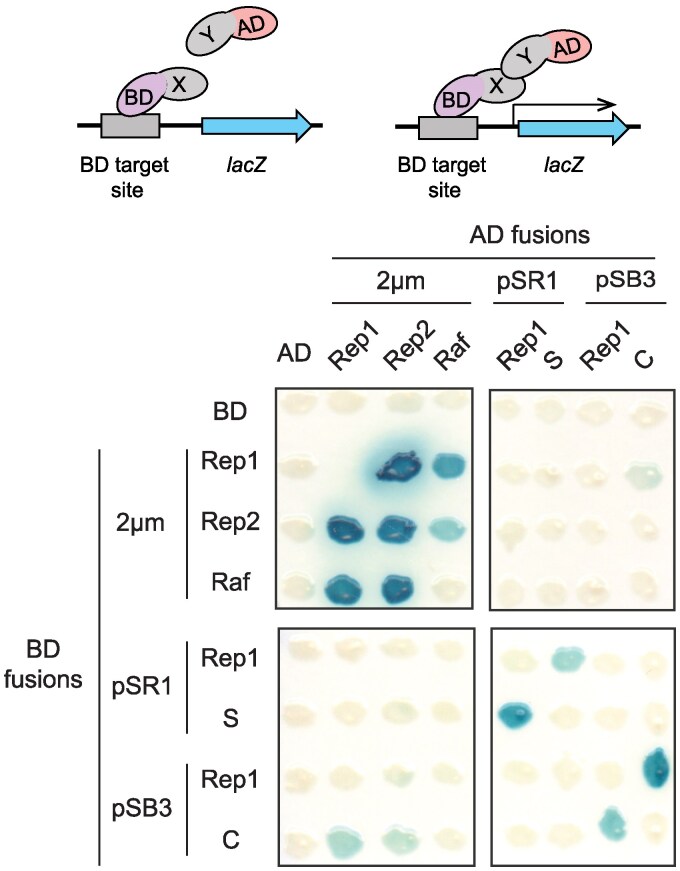
pSR1 and pSB3 partitioning proteins interact with their native partners in *S. cerevisiae* and pSB3 C also interacts with 2μm Rep1 and Rep2. A [*cir^0^*] *S. cerevisiae* two-hybrid reporter strain was co-transformed with two plasmids, one expressing the DNA-binding domain of LexA (BD), or BD fused to the plasmid protein shown at left, and the other expressing the Gal4 activation domain (AD), or the indicated plasmid protein fused to AD. Activation of the *lacZ* reporter gene, an indication of interaction between the proteins fused to AD and BD, respectively (X and Y in cartoon at top), was monitored using a filter assay with the substrate X-gal, which yields a blue precipitate when cleaved by β-galactosidase. Filters were imaged at 24 h.

As expected, 2μm Rep2 displayed strong two-hybrid interaction with Rep1, Raf and itself, as did 2μm Rep1 with Rep2 and Raf (Fig. 7) (Ahn et al. 1997; Scott-Drew and Murray 1998; Velmurugan et al. 1998; Sengupta et al. 2001; McQuaid et al. 2017). Neither pSR1 nor pSB3 Rep1 displayed self-association in this assay. Like 2μm Rep1, these Rep1 fusion proteins may need to be over-expressed from galactose-inducible promoters for their dimerization to be detected (Yang et al. 2004; McQuaid et al. 2017). Interaction of pSR1 Rep1 with pSR1 S protein and pSB3 Rep1 with pSB3 C protein was detected with no cross-reactivity between the products of these two *Z. rouxii* plasmids. This specificity may underlie the ability of the pSR1 and pSB3 plasmids to stably co-exist in *Z. rouxii* cells (Toh-e and Utatsu 1985). Unlike 2μm Rep2, neither the pSR1 S protein nor the pSB3 C protein displayed self-association in this assay. They may differ from Rep2 and not self-associate. Alternatively, the moieties fused to C and S may interfere with dimerization or, like Rep1, activation of the reporter gene above the background level may require expression of the fusion proteins at a higher level than provided here. *In vitro* baiting assays with purified proteins are needed to distinguish these possibilities.

### Amino-terminal domains of pSB3 Rep1 and C are required and sufficient for interaction

**Fig. 8.**
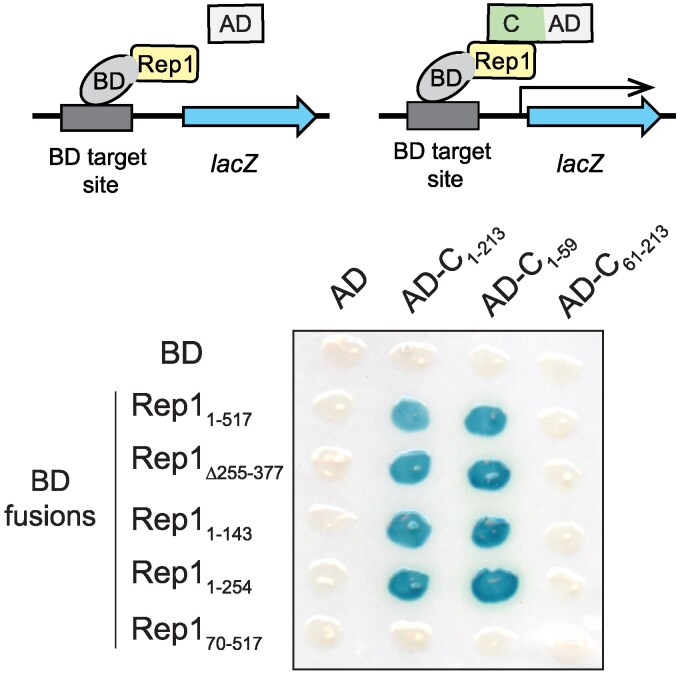
Amino-terminal domains of the pSB3 Rep1 and C proteins are sufficient and required for their association. A [*cir^0^*] *S. cerevisiae* two-hybrid reporter was co-transformed with two plasmids, one encoding AD, or AD fused to either full-length or truncated pSB3 C protein and the other encoding BD, or BD fused to the indicated version of pSB3 Rep1. Activation of *lacZ* reporter gene expression, an indication of interaction of the two fusion proteins (cartoon at top), was monitored as for Fig. 7. The filter imaged after 8 h is shown.

Intriguingly, the pSB3 C protein showed weak two-hybrid interaction with both 2μm Rep1 and Rep2, suggesting some limited structural resemblance to 2μm Rep2 and to Raf, both of which interact with 2μm Rep1 through small amino-terminal domains, residues 1 to 58 for Rep2 and 1 to 73 for Raf, respectively (McQuaid et al. 2017). To determine whether the pSB3 partitioning proteins interacted through similar domains to their 2μm counterparts, truncations of pSB3 Rep1 and C proteins, designed to closely match those of experimentally-determined domains in the 2μm proteins, with break points at positions that did not disrupt predicted secondary structure elements (McQuaid et al. 2017), were tested for their interaction with each other and with full-length partners using a two-hybrid assay (Fig. 8). The effect of deleting residues 255 to 377 from the internal non-conserved part of pSB3 was also assessed. (The region deleted was chosen to maximize alignment of the resulting protein with 2μm Rep1).

Like 2μm Rep1 and Rep2, amino-terminal regions of pSB3 Rep1 (1 to 143) and C (1 to 59) were found to be sufficient for their interaction while association was lost if either the first 69 residues of pSB3 Rep1, or the first 60 residues of pSB3 C were deleted. These results are the first demonstration that, despite differences in primary sequence identity, partitioning proteins of another member of this plasmid family interact through domains similarly positioned to the domains in 2μm Rep1 and Rep2 that are required and sufficient for their heterodimerization.

The internally-deleted pSB3 Rep1 protein also displayed two-hybrid interaction with pSB3 C that was as strong, if not stronger than that of the full-length pSB3 Rep1 (Fig. 8). Western blotting showed the deleted version had a higher steady-state level relative to the full length pSB3 Rep1 protein when these were expressed as AD fusions in the absence of their partner protein C (Supplementary Data Fig. 5). Deleting those codons might have improved either the efficiency of translation or the post-translational stability of the Rep1 fusion protein relative to the non-deleted version. The mitotic stability of pSB3-Kan and pSB3-flpΔ-Kan was also unaffected by deletion of the region encoding this internal portion of Rep1 suggesting that at least this part of the non-conserved region is not required for Rep1 partitioning function (Supplementary Data Fig. 4). The results are also consistent with the predicted structure for pSB3 Rep1 which shows this region not contributing to the other domains in the protein (Fig. 5).

### pSB3 gene upstream regions function in *cis* to increase plasmid inheritance in *Z. rouxii*

Functional analysis of the loci that function *in cis* to mediate partitioning of the 2μm-like family of plasmids, other than that of 2μm, has been limited. For three members of the family, plasmids pSR1, pKD1 and pKW1 (found in *Z. rouxii*, *K. lactis* and *L. waltii*, respectively), a DNA sequence that increases plasmid inheritance *in vivo* and acts as a plasmid partitioning locus in the native host has been identified (Jearnpipatkul et al. 1987a, 1987b; Bianchi et al. 1991) (A. Mereshchuk, unpublished results). These loci share no significant sequence identity with the repeats at the 2µm *STB* partitioning locus or with one another, and unlike 2µm *STB,* none have tandem-arrayed direct repeats (Utatsu et al. 1987; Volkert et al. 1989). The only features these loci share are the presence of A- and T-rich nucleotide stretches and being non-protein encoding.

**Fig. 9.**
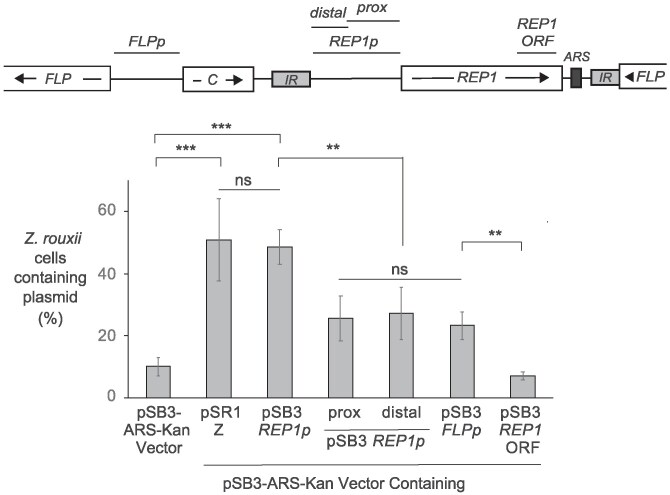
pSB3 plasmid gene promoter regions increase mitotic stability of the pSB3-ARS-Kan plasmid in *Z. rouxii. Z. rouxii* cells containing native pSB3 and pSR1 plasmids were transformed with pSB3-ARS-Kan or pSB3-ARS-Kan containing the pSR1 *cis*-acting partitioning sequence (*Z*), or part of pSB3. pSB3 sequences assessed are shown above. Transformants were cultured in selective medium (YPAD containing G418) and the percentage of cells carrying the *kanMX*-tagged plasmid was determined by a plating assay. Results represent the average (±StDev) (*n* = 5) (ns = not significant, **P < 0.01, *** *P* < 0.001).

To determine which sequence in pSB3 might function as the partitioning locus, each of the two pSB3 non-coding regions lying outside the *IR* sequences were cloned in plasmid pSB3-ARS-Kan, the plasmid that can replicate in *Z. rouxii*, conferring resistance to G418, but is partitioning-defective (Figs. 3 and 6). The test plasmids were assessed for mitotic stability in *Z. rouxii* cells that contained native pSB3 and pSR1 plasmids to supply the respective partitioning proteins *in trans* (Fig. 9). The plasmid containing the pSB3 *REP1* promoter region (*REP1p*) (extending from the start of the *REP1* ORF to the adjacent *IR* sequence) displayed a mitotic stability significantly higher than the empty vector pSB3-ARS-Kan and similar to that of pSB3-ARS-Kan containing the previously reported pSR1 *Z* partitioning locus (Araki et al. 1993). The pSB3 *FLP-ORF C* divergent promoter region (hereafter referred to as *FLPp*), also increased the mitotic stability relative to the empty vector pSB3-ARS-Kan, but to a lesser extent than *REP1p*. Attempts to further delineate the portion of *REP1p* responsible for the increased stability revealed that a 454-bp region immediately upstream of the *REP1* ORF or a 356-bp stretch further upstream and proximal to the *IR* were equally capable of providing a level of stability similar to that conferred by presence of the 755-bp pSB3 *FLPp* sequence, although neither was as effective as the complete 839-bp *REP1p* region. To rule out the possibility that the observed increase in mitotic stability might merely be due to recombination between the resident pSB3 plasmid and the pSB3 sequences inserted in the test plasmids, an internal stretch of the pSB3 *REP1* ORF was also cloned in pSB3-ARS-Kan. Mitotic stability of this plasmid was no better than that of the empty vector. The results suggest that both pSB3 promoter regions are capable of increasing plasmid inheritance independently of one another, possibly reflecting the ability of the pSB3 partitioning proteins to be recruited to these regions. If transcriptional regulation of pSB3 resembles that of the 2μm plasmid, the pSB3 Rep1 and C proteins may associate with both promoter regions to negatively regulate expression of the *REP1*, *FLP* and *C* genes when plasmid copy number becomes too high, in addition to their role in partitioning (Volkert and Broach 1986; Reynolds et al. 1987; Som et al. 1988). The higher stability conferred by the sequence extending from the *REP1* ORF to the *IR* (hereafter referred to as *PAR*) is consistent with this region being the *cis*-acting pSB3 partitioning locus, a location similar to that identified for the partitioning loci of the 2μm-like family plasmids, pSR1, pKD1 and pKW1 (Jearnpipatkul et al. 1987a, 1987b; Bianchi et al. 1991) (Mereshchuk, A., unpublished results).

### pSB3 partitioning protein recognition of the pSB3 *PAR* sequence in *S. cerevisiae*

**Fig. 10.**
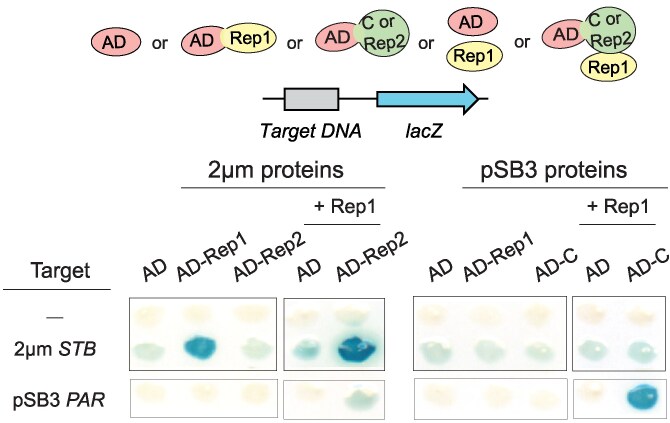
The pSB3 Rep1 and C proteins associate with the pSB3 *PAR* sequence in *S. cerevisiae* when co-expressed with their partner partitioning protein, and do not recognize the 2μm *STB* partitioning locus. *S. cerevisiae* yeast cells lacking native 2μm plasmid and containing the partitioning locus from the indicated yeast plasmid integrated in the genome upstream of a *lacZ* reporter gene, or no target sequence inserted (−), were transformed with a plasmid expressing AD, or the indicated 2μm or pSB3 protein fused to AD, either alone, or with a plasmid co-expressing their partner Rep1 protein (+Rep1). Activation of *lacZ*, an indication of association of the AD fusion protein with the target sequence, was assessed by a β-galactosidase filter assay.

The 2µm Rep1 and Rep2 proteins are both found associated with the plasmid *STB* partitioning locus *in vivo* (Velmurugan et al. 1998; Yang et al. 2004), with Rep2 requiring the presence of Rep1 for robust interaction (Pinder et al. 2013). To test whether the partitioning proteins encoded by pSB3 behaved similarly, the proteins were tested for their association with pSB3 *PAR* in a one-hybrid protein–DNA interaction assay (Fig. 10). When expressed in the absence of their partner protein, neither pSB3 AD fusion protein activated expression of the reporter gene, in contrast to 2μm Rep1 which, as previously reported, activated expression of the reporter downstream of *STB* in the absence of Rep2 (Yang et al. 2004; Pinder et al. 2013). However, when the pSB3 C fusion protein was co-expressed with its pSB3 Rep1 partner, the reporter gene downstream of pSB3 *PAR* was activated, indicating recruitment of the C protein AD fusion to the pSB3 *PAR* sequence. Like 2μm Rep2, pSB3 C may require Rep1 for stable association with the *PAR* sequence or, based on the inability of pSB3 Rep1 to activate the reporter in the absence of pSB3 C, a Rep1/C heterodimer may be needed for target recognition. Alternatively, in the absence of C the steady-state level of the large pSB3 Rep1 fusion protein might be too low to activate the reporter in this assay (Supplementary Data Fig. 5).

### Partitioning loci from pSR1 and pSB3 cannot functionally substitute for 2μm *STB*

Interestingly, the one-hybrid results (Fig. 10) indicate 2µm Rep2, when co-expressed with 2µm Rep1, in addition to being targeted to *STB*, also associated weakly with the pSB3 *PAR* sequence, suggesting that features required for recognition of the locus by the partitioning proteins might be conserved between 2µm *STB* and pSB3 *PAR*. This raised the possibility that the pSB3 partitioning locus might at least partially be able to substitute for the 2μm *STB* locus and function with 2 μm Rep1 and Rep2 to increase the inheritance of a 2μm plasmid in *S. cerevisiae.* This was assessed by replacing the *STB* repeat region of a 2μm-based plasmid with the pSB3 *PAR* sequence. However, the plasmid was found to be as unstable as the parental 2μm plasmid lacking the *STB* repeats (Supplementary Data Fig. 6). This shows that despite weak recognition of pSB3 *PAR* by the 2μm Rep1/Rep2 heterodimer, the 2μm proteins only function effectively with 2μm *STB*, suggesting the lack of sequence identity between the 2µm and pSB3 partitioning loci might reflect a difference in the sequence specificity of their respective partitioning proteins.

### pSB3 Rep1 and C associate with pSB3 promoter regions but possibly less avidly than with *PAR*

**Fig. 11.**
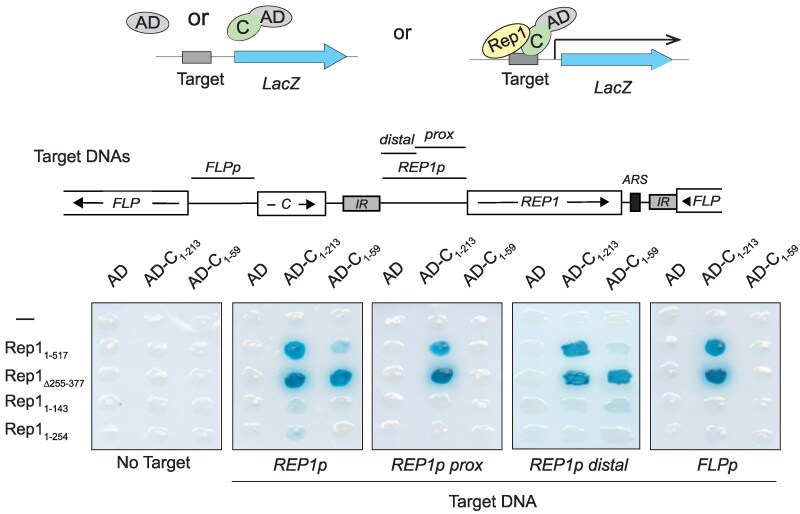
Deletion of the pSB3 Rep1 carboxy-terminal domain, but not the internal non-conserved region, impairs Rep1-dependent recruitment of pSB3 C to the pSB3 promoter regions, while protein C lacking the 154 carboxy-terminal residues retains Rep1-dependent association only with the distal *REP1* promoter region. AD or the indicated AD-pSB3 C fusion protein was expressed in [*cir^0^*] *S. cerevisiae* cells containing a target DNA sequence integrated in the genome upstream of a *lacZ* reporter gene (*diagram of assay and position of pSB3 sequences used as targets are shown at top*). Full length or truncated versions of pSB3 Rep1 were co-expressed in the cells as indicated at left. Recruitment of the AD fusion to the target was monitored as in Fig. 10.

For the 2µm plasmid the Rep proteins mediate partitioning when bound at *STB* (Yang et al. 2004), but also negatively regulate plasmid copy number amplification by associating with the *FLP* gene promoter and repressing transcription (Volkert and Broach 1986; Reynolds et al. 1987; Som et al. 1988). The ability of the pSB3 protein-encoding gene promoter regions to increase mitotic stability of an *ARS*-only plasmid in *Z. rouxii* (Fig. 9), suggested the pSB3 partitioning proteins might likewise associate with the pSB3 *FLP-ORF C* divergent promoter region (*FLPp*). To test this and to try to gain insight into the nature of the association, full-length and truncated versions of the pSB3 Rep1 and C proteins were assessed for one-hybrid interaction with pSB3 promoter regions (Fig. 11).

As observed in Fig. 10, when the pSB3 C AD fusion protein was co-expressed with pSB3 Rep1, the reporter downstream of *PAR* (full *REP1* upstream region extending to the *IR*) was activated (Fig. 11, second panel). Similar activation was observed for reporters placed downstream of *FLPp*, and for either of two subsections of *PAR*, one proximal, and the other distal to the *REP1* ORF. No activation was observed in the absence of Rep1 co-expression indicating that like 2μm Rep2, Rep1 was needed for efficient recruitment of pSB3 C to these sequences.

A similar pattern, but with much stronger activation of the reporter genes, was observed when full-length pSB3 C was expressed with the internally deleted version of pSB3 Rep1 (amino acids 255 to 377 deleted). Stronger activation may reflect a higher steady state level of the deleted version relative to full-length Rep1 (517 amino acids) (Supplementary Data Fig. 5) and demonstrates the deleted region does not contain elements required for Rep1 recruitment of C to *PAR*. In contrast, when pSB3 C was co-expressed with either of two carboxy-terminally truncated versions of pSB3 Rep1 (Rep1_1-143_ and Rep1_1-254_), reporter gene expression was virtually abolished for all test target DNAs. These same Rep1 truncations interacted strongly with full-length pSB3 C and C_1-59_ in the two-hybrid assay (Fig. 8), making it unlikely that low steady state levels of the Rep1 truncations or loss of C interaction were responsible for Rep1 failing to efficiently recruit C to the pSB3 sequences here. The results suggest that the deleted carboxy-terminal portion of pSB3 Rep1 contains sequence elements needed for Rep1 recognition of these loci. For the *PAR* reporter, but not the others, we did note a slight activation above background when the full-length pSB3 C AD fusion was expressed with the carboxy-terminally-truncated versions of Rep1 (Fig. 11, second panel), an activation not observed for the C_1-59_ AD fusion expression. The truncated C fusion may not be present at a level high enough for the interaction to be detected. Alternatively, this weak activation might represent the carboxy-terminal domain of C being required for the C protein to bind *PAR* independently of Rep1 target recognition. This region does contain a basic sequence motif also found in the carboxy-terminal domains of 2μm Rep2 and other Rep2-equivalent partitioning proteins encoded by other 2μm family plasmids (McQuaid et al. 2017). For 2μm Rep2, the 65-amino acid carboxy-terminal domain containing this motif has been shown to have DNA-binding activity *in vitro* (McQuaid et al. 2017) and to be sufficient, even in the absence of Rep1, for promoting inheritance of a replication-competent plasmid lacking *STB* if artificially tethered to the plasmid (Mereshchuk et al. 2022). Whether this motif contributes to DNA-binding activity of pSB3 C or mediates C interaction with a host factor remains to be demonstrated.

Taken together, the one-hybrid results indicate that in cells expressing the internally deleted version of pSB3 Rep1 (which is likely present at a higher level than the undeleted version), the carboxy-terminal portion of C is not required for C to be recruited to the *IR*-proximal portion of *PAR* but is required for robust association with the *FLP* promoter and the immediate upstream *REP1* promoter region. This suggests different affinities between the promoter-proximal sites relative to those in the *IR*-proximal portion of *PAR*. Quantitative chromatin immunoprecipitation and mutational analysis will be needed to assess this further and to determine which sequence elements are required at these distinct sites for Rep1 and C association.

## Discussion

The near chromosome-level inheritance of 2μm family plasmids is remarkable given that these extra-chromosomal genetic elements confer no benefit to the host yeast cells. Equally surprising is the minimal detriment the plasmids impose despite being present at high copy number. For the founding member of the group, the 2μm circle of *S. cerevisiae*, association of the 2μm Rep1 and Rep2 proteins with the plasmid *STB* locus enables plasmid copies to be equally partitioned at cell division (Ahn et al. 1997; Scott-Drew and Murray 1998; Yang et al. 2004). Rep1/Rep2 heterodimers also associate with the plasmid gene promoters, negatively regulating plasmid amplification and preventing Rep protein expression from reaching toxic levels (Volkert and Broach 1986; Reynolds et al. 1987; Som et al. 1988; Scott-Drew and Murray 1998; Dobson et al. 2005). Although the outcome of Rep protein interactions at these loci has been established, the nature of the associations and how each contributes remains unclear. In this study, 2μm-like plasmids from other budding yeast species were found to encode Rep1 proteins more similar to 2μm Rep1 than earlier DNA sequencing had suggested (Fig. 4, Supplementary Data Fig. 2 and Supplementary Data Table 4). The Rep1 protein of *Z. rouxii* plasmid pSB3 was also found to resemble 2μm Rep1 with respect to the domain required for interaction with its partner partitioning protein C, and in enabling C recruitment to the pSB3 partitioning locus and plasmid gene promoter regions (Figs. 7, 10 and 11). These results, combined with protein modeling (Fig. 5, Supplementary Data Fig. 2 and Supplementary Data Table 4) suggest Rep1 protein function and structure have been conserved across the currently known members of the 2μm family of plasmids.

### Partitioning protein interactions

Previous studies have shown 2μm Rep1 and Rep2 interact *in vitro* and *in vivo* through amino-terminal domains in each (residues 1 to 99 for Rep1 and 1 to 58 for Rep2), and that both self-associate, through residues 1 to 129 for Rep1, and 58 to 231 for Rep2 (Ahn et al. 1997; Scott-Drew and Murray 1998; Velmurugan et al. 1998; Sengupta et al. 2001; McQuaid et al. 2017). Three-dimensional modeling of 2μm Rep1 and Rep2 is consistent with this experimental data, predicting the amino-terminal regions of each represent an independently folded domain, separated from the carboxy-terminal portion of the protein by a variable-length linker (Fig. 5 and Supplementary Data Fig. 2).

Comparison of the Rep1 proteins encoded by different family members identified two amino acid motifs within the amino-terminal domain (Supplementary Data Fig. 1). Mutations within these motifs in 2μm Rep1 (Y50A, A50D and V78K) have been shown to abolish Rep1 interaction with Rep2 without affecting association with *STB* (Yang et al. 2004). These elements could therefore be direct targets for Rep2 recognition, or alternatively, given 2μm Rep2 did not interact with the Rep1 protein encoded by *Z. rouxii* plasmid pSR1 which also contains these motifs (Fig. 7), these elements may be conserved because they are critical for the structural integrity of the amino-terminal domain. Mutation of the motifs might have affected the protein structure significantly enough to compromise recognition of the domain by the Rep1 partner partitioning protein.

In this study, amino-terminal domains of the *Z. rouxii* pSB3 plasmid partitioning proteins Rep1 and C were also found to be sufficient for their interaction (Fig. 8). Surprisingly, pSB3 C also displayed two-hybrid association with 2μm Rep1 and Rep2 (Fig. 7), suggesting C might retain some structural feature of the Rep2 amino-terminal region despite lack of amino acid sequence similarity between the two (McQuaid et al. 2017).

Unlike 2μm Rep1 and Rep2, neither the pSB3 nor the pSR1 partitioning proteins displayed self-association in two-hybrid assays in this study (Fig. 7). Whether this represents a distinct difference from the 2μm proteins, or merely, some feature of the assay system that prevented the interaction from being detected is unknown. For pSB3 Rep1, steady-state levels of the protein when expressed in the *S. cerevisiae* two-hybrid reporter strain in the absence of its partner C protein might have been too low to allow self-association to be detected (Supplementary Data Fig. 5). Another difference from 2μm Rep1 was the requirement for co-expression of pSB3 C with pSB3 Rep1 for the one-hybrid association of Rep1 or C with the pSB3 *PAR* sequence to be detected (Fig. 10). Again, this could reflect lower levels of the two proteins in this heterologous yeast host but could indicate a difference in how the pSB3 sequence, which lacks the tandem repeats of the 2μm *STB* repeat array, is bound.

### How do the 2μm family partitioning proteins associate with plasmid DNA loci *in vivo*?

**Fig. 12.**
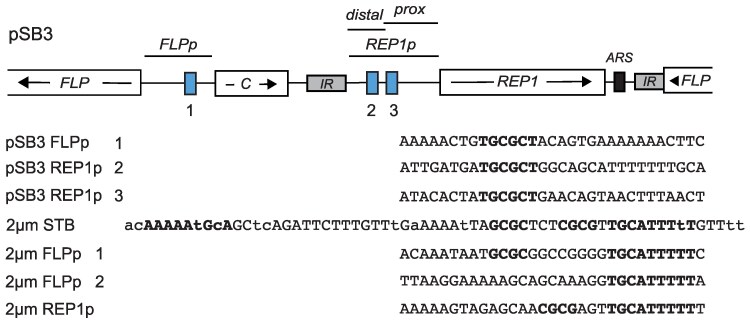
Comparison of pSB3 and 2μm plasmid sequences required for partitioning protein association. Position of regions in pSB3 that increase *ARS* plasmid mitotic stability in *Z. rouxii* and were recognized by the pSB3 Rep1 and C proteins in one-hybrid assays are shown (*top*), with potential sequences targeted at those sites by the partitioning proteins shown (*below*) aligned with those of the 2μm plasmid. TGCGCT motifs in pSB3, and GCGC, TGCATTTTT and CGCG elements in 2μm are in bold. For 2μm *STB*, the sequence of a single Type II repeat is shown with lower-case letters indicating positions where a different residue is found in two other repeats in the five-repeat array.

An outstanding puzzle is the nature of the complex formed between the 2μm family partitioning proteins and the DNA sequence at their respective partitioning loci. The carboxy-terminal portion of Rep1 (residues 130 to 373) is sufficient for recruitment to *STB* in a one-hybrid assay, with mutagenesis studies identifying amino acid substitutions in this part of 2μm Rep1 that led to loss of *STB* association without abolishing Rep2 interaction (Sengupta et al. 2001; Yang et al. 2004; Pinder et al. 2013) (Fig. 4). AlphaFold modeling of 2μm Rep1 places these residues within alpha helices or beta-strands that are components of the two domains predicted for this carboxy-terminal two-thirds of the protein and for the corresponding portion of pSB3 Rep1 (Fig. 5). Some *in silico* models suggest this portion of 2μm Rep1 might be able to directly recognize the *STB* repeat DNA with residues K297, Y301, V316, Y317, and R318 contacting a TGCATTTTT motif and an adjacent CGCG element (Fig. 12) (data not shown). This would be consistent with experimental data showing K297Q and Y317I mutations abolish Rep1-*STB* association in a one-hybrid assay (Yang et al. 2004), and with mutations in those two *STB* motifs disrupting plasmid partitioning (McQuaid 2015; McQuaid et al. 2019b).

Despite *in silico* modeling suggesting the 2μm Rep1 protein should be capable of directly binding the 2μm *STB* DNA sequence, 2μm Rep1 has not been demonstrated to have DNA-binding activity *in vitro* (Hadfield et al. 1995) (Dobson, unpublished results). This might be a technical issue; the majority of the 2μm Rep1 protein is found in the insoluble fraction whether expressed in bacteria or when purified from yeast protein extracts. Even when solubilized, the purified Rep1 protein is observed to form dimers that persist even under denaturing gel conditions with the amino-terminal 65 residues sufficient for this self-association (McQuaid et al. 2017). The conformation of the carboxy-terminal portion of Rep1 in these complexes may differ from the configuration required for DNA binding. Alternatively, Rep1 might require association with a host protein to assume the appropriate conformation. Evidence for such a host factor comes from surface plasmon resonance assays where bacterially-expressed Rep1 and Rep2 were found to associate with *STB* DNA *in vitro* independently of one another, but only when a urea-solubilized protein extract from a [*cir^0^*] yeast was present (Hadfield et al. 1995). Rep1 might need the *STB* repeat DNA organized into a particular conformation by the bound host factor or require interaction with the host factor to bind at *STB*.

### Comparison of plasmid sequences recognized by the pSB3 and 2μm partitioning proteins

Although sequence elements required for Rep protein recognition of the 2μm *STB* partitioning locus have been identified, those recognized by the partitioning proteins of other members of the 2μm plasmid family have not. For those where the partitioning locus is known (Bianchi et al. 1991; Araki et al. 1993), the sequences share little in common other than the presence of A- and T-tracts, that by their stiffness, would impose constraints on wrapping the DNA into nucleosomes (Lorch et al. 2014; Schlichter et al. 2020). Spacing between these tracts varies between loci with possibly the most consistent feature being juxtaposition of a more AT-rich stretch next to a comparatively GC-rich region in each (data not shown). Here, investigation of the sequences required for partitioning of the pSB3 plasmid identified two regions upstream of the pSB3 *REP1* gene and the promoter region of the *FLP* gene as being able to increase *ARS* plasmid mitotic stability in *Z. rouxii* cells where the native plasmid was present to supply the Rep1 and C proteins *in trans* (Fig. 9). One-hybrid assays indicated pSB3 Rep1 was able to recruit the C protein to each of these (Fig. 11). Comparison of the three regions identified a TGCGCT motif in each with only the sequence closest to the *REP1* ORF lacking a nearby A- or T-tract (Fig. 12). Preliminary modeling suggested pSB3 Rep1 might contact residues within this motif in each with additional contacts in flanking sequences that varied between the three sequences (data not shown). Further modeling and mutational analysis are needed to determine whether these represent *bona fide* targets *in vivo*. Interestingly, the consensus sequence for 2μm *STB* repeats also contains a GCGC element. Although mutation of this element has not investigated experimentally, Rep2 was recruited by 2μm Rep1 to the full pSB3 *REP1* upstream region (*PAR*) containing both pSB3 Rep1/C association sites (Fig. 10), suggesting some feature in common with the *STB* repeat.

### Role for pSB3 partitioning proteins in transcriptional regulation and plasmid copy number control

Although we did not directly test whether the pSB3 Rep1/protein C heterocomplex has the transcriptional repressor activity of 2μm Rep1/Rep2, the one-hybrid association of pSB3 Rep1 and protein C with the pSB3 *FLP* and *REP1* gene promoter regions (Fig. 11) is akin to the interaction of 2μm Rep1/Rep2 with the upstream regions of the 2 μm plasmid genes (Yang et al. 2004; McQuaid et al. 2017, 2019b; Rizvi et al. 2017). The *FLP* promoter contains two TGCATTTTT Rep1-recognition targets and the *REP1* promoter one. Deletion or mutation of the TGCATTTTT most distal to the *FLP* ORF (sequence 2 in Fig. 12), but not that of the other copy, resulted in hyper-amplification of a Flp-competent 2μm plasmid and a nibbled colony phenotype consistent with this TGCATTTTT being the functional target for Rep protein-mediated repression (Pinder 2011). The sequences flanking the TGCATTTTT elements in the 2μm gene promoters and those flanking the TGCGCT elements in pSB3 are aligned for comparison in Fig. 12. The pSB3 *REP1* promoter-distal fragment did contain a TTTTTGCA motif but whether this contributes to plasmid stability or repression of *REP1* gene expression remains to be determined.

### The role of 2μm Rep2 and other Rep1 partner proteins in plasmid partitioning

In contrast to lack of demonstrable DNA-binding activity for 2μm Rep1, the C-terminal 65 amino acids of 2μm Rep2 has been shown to bind DNA *in vitro* (Sengupta et al. 2001) and when artificially tethered to a plasmid that could replicate but not partition in yeast, plasmid inheritance was increased, even in the absence of Rep1 (Mereshchuk et al. 2022). Mutation of a basic motif in this sequence led to loss of this Rep1-independent partitioning activity but did not affect Rep2 DNA-binding activity (Mereshchuk et al. 2022). As this motif is also found in in the C-terminal region of pSB3 C and other Rep1 partner partitioning proteins of the 2μm plasmid family (McQuaid et al. 2017), this domain might directly contribute to recognition of the respective plasmid partitioning sequences or be involved in capture of segregating chromosomes by the partitioning protein complexes bound at those partitioning loci. A role for the pSB3 C protein C-terminal region in recognizing plasmid target sequences was indicated by results of one-hybrid assays in which pSB3 Rep1-dependent association of C with the pSB3 *FLP* promoter and a proximal portion of the *REP1* promoter was lost when the C-terminal 154 amino acids of C was deleted, while association with the distal region of the *REP1* promoter was retained (Fig. 11). This suggested that C-terminal region of C might be assisting binding at those potentially less optimal sites.

### Host factors participating in partitioning of the 2μm plasmid family

Association of 2μm Rep1 and Rep2 with the *STB* partitioning locus aggregates the multiple copies of the plasmid into a small number of nuclear foci that persist throughout the cell cycle (Velmurugan et al. 2000; Scott-Drew et al. 2002). Equal partitioning of these clustered plasmids between the mother and daughter cell at mitosis depends on a variety of host factors, many of which are required for chromosome segregation, consistent with the plasmid relying on chromosome attachment to ensure equal partitioning at cell division (for review, see Rizvi et al. 2018; Sau et al. 2019).

Among these factors, some are recruited to *STB* by the 2μm Rep proteins suggesting a more direct role (Ma et al. 2013; Rizvi et al. 2018). These include the nuclear motor protein Kip1 (Cui et al. 2009), the centromere-specific histone H3 (Cse4 in yeast) (Hajra et al. 2006), and cohesin (Mehta et al. 2002) but kinetochore proteins are not recruited, and the plasmids are not captured by the plus end of microtubules (Mehta et al. 2002, 2005). In addition, the RSC2 complex, one of two related chromatin remodelers in *S. cerevisiae*, the other being the RSC1 complex (Cairns et al. 1999), is also localized to *STB* (Wong et al. 2002). Several lines of evidence provide significant support for the RSC2 complex being integral to the Rep protein partitioning complex formed at *STB* and also potentially bridging interaction between the Rep proteins at *STB* and sites on chromosomes bound by RSC2 (Ma et al. 2023). In tandem-affinity purification experiments, Rep2 pulled down several RSC components including Rsc3 and Rsc2, the latter being the subunit that distinguishes the RSC2 complex from the RSC1 complex which includes Rsc1 rather than Rsc2 (Cairns et al. 1999). Deletion of the gene encoding Rsc2, but not of that encoding Rsc1, altered nuclease-sensitivity of *STB* chromatin and impaired 2μm plasmid inheritance (Wong et al. 2002). Of relevance here, the DNA-binding component of both the RSC1 and RSC2 complexes, a Rsc3/Rsc30 heterodimer, recognizes a CGCG motif (Badis et al. 2008), an element found in the *STB* consensus repeat sequence. This raises the possibility that Rsc3, which temporal experiments suggest would associate with *STB* before the Rep proteins when the partitioning complex is reassembled during the G1-to-S phase transition (Ma et al. 2013), might be the host factor Rep1 and Rep2 required for *STB* association to be detected in gel shift and plasmon resonance assays (Hadfield et al. 1995). It will be of interest to determine whether the other 2µm family plasmids which are found in yeast lineages that did not undergo the whole genome duplication that led to two versions of the RSC complex in *Saccharomyces* (Fig. 1), or that lack a CGCG motif in their partitioning sequence (pSM1), are similarly dependent on this chromatin remodeler for their inheritance or whether this represents a specialized adaption linked to the unusual tandem array repeats at the 2μm *STB* partitioning locus.

In addition to host factors recruited to *STB*, Rep1 and Rep2 are subject to host-mediated phosphorylation (Pinder 2011) and post-translational modification with the host small-ubiquitin-related modifier SUMO (Pinder et al. 2013). Sumoylation-deficient mutants of Rep1 and Rep2 were impaired for association with *STB* and normal co-localization of both Rep proteins with the punctate nuclear plasmid foci was also lost when Rep1 was sumoylation-deficient (Pinder et al. 2013). Whether phosphorylation or sumoylation is needed to alter Rep protein conformation or allow the Rep proteins to interact with a chromosome-bound factor is unknown. The pSB3 Rep1 and C partitioning proteins may also be sumoylated as both displayed two-hybrid interaction with a conjugatable form of yeast SUMO (Smt3) when expressed without their partner in *S. cerevisiae* (Dobson, unpublished observations).

Locations reported for the position of the 2μm plasmid foci in the yeast nucleus have differed. Fluorescently-tagged 2μm plasmid clusters have been observed to reside close to the spindle pole bodies or near centromeric regions of chromosomes (Velmurugan et al. 2000; Mehta et al. 2005) or near telomeric regions in meiosis (Sau et al. 2014, 2019). ChIP-seq and CUT&Tag techniques identified tRNA and snoRNA genes as well as centromere and telomeric regions as sites enriched for Rep protein and RSC2 complex association (Ma et al. 2023). Recently, proximity ligation mapping has shown Rep protein/*STB*-dependent preferential tethering of 2μm plasmid copies to several chromosomal regions with low transcriptional activity with contacts being lost when transcription at those sites was activated or nucleosome H4 basic tails were altered, suggesting the Rep proteins complexed at *STB* might link the plasmid to chromosomal sites with a particular chromatin organization (Girard et al. 2025). While the precise nature of this chromosome tethering remains to determined, the efficient partitioning of pSB3 in multiple yeast species indicates that any host factors this member of the 2μm family utilizes to attach to segregating host chromosomes are likely to be conserved across these species. This may help focus the search for those factors. Active partitioning of the pSB3 plasmid and the conservation of the pSB3 partitioning protein interactions in *S. cerevisiae* now allows mix and match experiments between 2µm and pSB3 plasmid elements that may help identify the protein residues, DNA features and host proteins required for the maintenance of the 2µm plasmid family.

### Evolutionary considerations

The restricted distribution of 2μm family plasmids in a small number of yeast species of the Saccharomycetaceae lineage, the similarity of their organization and the conservation of the Flp recombinase, and now of the Rep1 partitioning protein encoded by each, all support the plasmids having arisen from a common ancestor, despite their lack of DNA sequence identity (Volkert et al. 1989; Blaisonneau et al. 1997). The possibility has been raised that the chromosomal capture of the *cis*-acting partitioning component of such an ancestral 2μm family plasmid may have instigated the change from the epigenetic centromere found in other fungi to the unusual point centromere (*CEN*) of the Saccharomycetaceae lineage (Malik and Henikoff 2009). Subsequent competition between the plasmid and chromosomal *CEN* sequences for plasmid partitioning proteins may have driven rapid evolution of the plasmid partitioning components (Malik and Henikoff 2009). It seems most plausible that as budding yeast species evolved from the ancestral Saccharomycetaceae host, the 2μm family plasmid initially present subsequently also diverged in concert with the host cells.

### Potential biotechnological relevance

The finding that pSB3-based plasmids are efficiently replicated and partitioned in multiple budding yeast species suggests pSB3 might prove useful as a basis for high-copy number vectors for biotechnological applications. Industrial processes often involve more extreme environmental conditions that may not be well tolerated by *S. cerevisiae*, the 2µm circle host, but which might be possible for the more osmophilic pSB3 native host *Z. rouxii* (Toh-e et al. 1984; Utatsu et al. 1987). In that regard, it may be merely fortuitous that pSB3 contains a sequence that functions as an efficient origin of DNA replication in more budding yeast species than the *S. cerevisiae* 2µm circle *ARS,* contributing to the high mitotic stability observed here for the pSB3-based plasmids *(*Fig. 6). This may not be so surprising as DNA replication origins in *Z. rouxii* and *L. waltii* have been reported to have *ARS* consensus sequences that differ from that of *S. cerevisiae* (Araki and Oshima 1989; Di Rienzi et al. 2012). pSB3-based vectors might also present an advantage even in *S. cerevisiae* if they can be maintained at a high copy number without triggering the toxicity associated with combined over-expression of the 2µm Rep1 and Rep2 partitioning proteins (Reynolds et al. 1987; Dobson et al. 2005). Longer periods of propagation are needed to determine whether the plasmids can be maintained indefinitely in the absence of selection and whether introducing gene expression cassettes at the neutral site we have identified downstream of the pSB3 C gene transcription termination sequence alters plasmid maintenance.

### Significance of Rep1 protein conservation

This study has corrected errors in the previously reported Rep1 protein sequences encoded by four of the eight known 2µm family plasmids, allowing identification of Rep1 sequence motifs and prediction of structural features that have been conserved between 2µm plasmid family members. Such conservation suggests these elements are critical for Rep1 function. Analyzing differences between the Rep1 proteins might reveal how the specificity of the interaction with their partner partitioning protein and targeting to their particular partitioning locus DNA sequence is accomplished.

Overall, this research broadens the understanding of the maintenance mechanism of the 2µm family of plasmids and is the first experimental analysis of the interactions of the partitioning proteins encoded by a member other than the 2µm circle. Further studies are still needed to understand how this selfish DNA plasmid family manages its faithful maintenance.

## Supplementary Material

jkag012_Supplementary_Data

## Data Availability

The authors affirm that all data necessary for confirming the conclusions of the article are present within the article, figures, and tables. Plasmid sequence data are available at GenBank; accession numbers are provided in the article and the Supplementary Data file. Strains and plasmids are available upon request. Supplemental material available at G3 online.
