# Supplementary material for: pSB3, a member of the 2-micron family of plasmids, replicates and is efficiently partitioned in multiple budding yeast species
Source: G3 (Bethesda). 2026 Feb 18;16(8):jkag012. doi: 10.1093/g3journal/jkag012 (PMC13439956; doi:10.1093/g3journal/jkag012)
Supplement: Supplementary file 1 [file jkag012_supplementary_data.pdf]

Supplementary Data

pSB3, a member of the 2-micron family of plasmids, replicates and is efficiently partitioned in multiple budding yeast species

Anastasiia Mereshchuk, Joyce S.K. Chew, and Melanie J. Dobson*
Department of Biochemistry and Molecular Biology, Dalhousie University, Halifax, Nova Scotia, B3H 4R2, Canada

* To whom correspondence should be addressed. Tel: 902-494-6436; Fax: 902-494-1355; Email: dobson@dal.ca

Table S1. Yeast used in the study

S. cerevisiae strain	Relevant Genotype	Source
MD83/1c	*MATα ade2-1 his3-11,15 trp1-1 leu2-3,112 ura3-1 [cir^0]*	(1)
MD83/1c-no UAS-lacZ	*URA3::GALp(ΔUAS)-lacZ [cir^0]*	This study
MD83/1c-2μm-STB-P-lacZ	*URA3::2μm STB-proximal-GALp(ΔUAS)-lacZ [cir^0]*	This study
CT/MD/3a	*MATa his3 trp1 leu2-3,112 ade2-1 ura3 met URA3::(lexA$_{(op8)}$)-lacZ [cir^0]*	(2)
AG8/5	*MATa ade2Δ::URA3 his3-11,15 leu2-3, 112 trp1-1 ura3-1 [cir^0]*	(2)
JP49/6b	*MATa ade2-1 his3-11,15 leu2-3,112 trp1-1 ura3-1 [cir^0]*	(2)
JP49/6b-no UAS-lacZ	*URA3::GALp(ΔUAS)-lacZ [cir^0]*	This study
JP49/6b-pSR1-Z-lacZ	*URA3::pSR1 Z-GALp(ΔUAS)-lacZ [cir^0]*	This study
JP49/6b-pSB3-PAR-lacZ	*URA3::pSB3 PAR-GALp(ΔUAS)-lacZ [cir^0]*	This study
JP49/6b-pSB3-FLPp-lacZ	*URA3::pSB3 FLPp-GALp(ΔUAS)-lacZ [cir^0]*	This study
JP49/6b-pSB3-REP1-ORF-lacZ	*URA3::pSB3 REP1-ORF-GALp(ΔUAS)-lacZ [cir^0]*	This study
JP49/6b -pSB3-REP1p-prox	*URA3::pSB3 REP1p-proximal-GALp(ΔUAS)-lacZ [cir^0]*	This study
JP49/6b -pSB3-REP1p-distal	*URA3::pSB3 REP1p-distal-GALp(ΔUAS)-lacZ [cir^0]*	This study
Other yeast species		
Lachancea waltii (previously classified as *Kluveromyces waltii)*	*[pKW1]*	CBS 6430 (NRRL Y-8285) (3)
Torulaspora delbrueckii	*[cir^0]*	Biodiva™ TD291, Scott Laboratories
Zygosaccharomyces bailii	Wild isolate strain EV1 *[pZB1]* [a]	Dobson lab yeast collection
Zygosaccharomyces rouxii (previously classified as *Zygosaccharomyces bisporus)*	*[pSB3, pSR1]*	ATCC 56076 (4)

[a] pZB1 (Genbank accession PX275371) is a variant of plasmid pSB2 (Dobson, unpublished results) (5) (6).

Table S2. Plasmids used in this study

Plasmid Name	Features	Source
pJL638	*URA3 GALp(ΔUAS)-lacZ*	(7)
pJL638-pSR1-Z	*URA3 pSR1 Z-(2401-2911)-GALp(ΔUAS)-lacZ*	This study
pJL638-pSB3-FLPp	*URA3 pSB3 FLPp-(1247-2001)-GALp(ΔUAS)-lacZ*	This study
pJL638-pSB3-PAR	*URA3 pSB3 REP1p-(4989-5827)-GALp(ΔUAS)-lacZ*	This study
pJL638 -pSB3 REP1p-proximal	*URA3 pSB3 REP1p-prox-(4989-5517)-GALp(ΔUAS)-lacZ*	This study
pJL638-pSB3-REP1p-distal	*URA3 pSB3 REP1p-distal-(5472-5827)-GALp(ΔUAS)-lacZ*	This study
pJL638-pSB3-REP1p-581	*URA3 pSB3 REP1p-(5036-5616)-GALp(ΔUAS)-lacZ*	This study
pGAD424	*LEU2 2μm ADH1p-GAL4$_{AD}$*	Clontech
pGADREP1	*LEU2 2μm ADH1p-GAL4$_{AD}$-2μm REP1*	(8)
pGADREP2	*LEU2 2μm ADH1p-GAL4$_{AD}$-2μm REP2*	(8)
pGADRAF	*LEU2 2μm ADH1p-GAL4$_{AD}$-2μm RAF*	(9)
pGAD-pSR1-REP1	*LEU2 2μm ADH1p-GAL4$_{AD}$ -pSR1 REP1*	This study
pGAD-pSR1-S	*LEU2 2μm ADH1p-GAL4$_{AD}$ -pSR1 S*	This study
pGAD-pSB3-REP1	*LEU2 2μm ADH1p-GAL4$_{AD}$ -pSB3 REP1*	This study
pGAD-pSB3-REP1Δ123	*LEU2 2μm ADH1p-GAL4$_{AD}$ -pSB3 REP1$_{1-254/378-517}$*	This study
pGAD-pSB3-C	*LEU2 2μm ADH1p-GAL4$_{AD}$ -pSB3 C*	This study

Plasmid Name	Features	Source
pGAD-pSB3-C_{1-59}	*LEU2 2μm ADH1p-$GAL4_{AD}$ -pSB3 C_{1-59}*	This study
pGAD-pSB3-C_{61-213}	*LEU2 2μm ADH1p-$GAL4_{AD}$ -pSB3 C_{61-213}*	This study
pSH2-1	*HIS3 2μ m ADH1p-$LexA_{BD}$*	(10)
pSHREP1	*HIS3 2μm ADH1p- $LexA_{BD}$ -2μm REP1*	(8)
pSHREP2	*HIS3 2μm ADH1p- $LexA_{BD}$ -2μm REP2*	(8)
pSHRAF	*HIS3 2μm ADH1p- $LexA_{BD}$ -2μm RAF*	(9)
pSHpKW1-REP1	*HIS3 2μm ADH1p- $LexA_{BD}$ -pKW1 REP1*	This study
pSHpSR1-REP1	*HIS3 2μm ADH1p- $LexA_{BD}$ -pSR1 REP1*	This study
pSHpSR1-S	*HIS3 2μm ADH1p- $LexA_{BD}$ -pSR1 S*	This study
pSHpSB3-REP1	*HIS3 2μm ADH1p- $LexA_{BD}$ -pSB3 REP1*	This study
pSHpSB3-$Rep1_{1-254/378-517}$	*HIS3 2μm ADH1p- $LexA_{BD}$ - pSB3 $REP1_{1-254/378-517}$*	This study
pSHpSB3-$Rep1_{1-143}$	*HIS3 2μm ADH1p- $LexA_{BD}$ -pSB3 $REP1_{1-143}$*	This study
pSHpSB3-$Rep1_{1-254}$	*HIS3 2μm ADH1p- $LexA_{BD}$ -pSB3 $REP1_{1-254}$*	This study
pSHpSB3-$Rep1_{70-517}$	*HIS3 2μm ADH1p- $LexA_{BD}$ -pSB3 $REP1_{70-517}$*	This study
pSHpSB3-C	*HIS3 2μm ADH1p- $LexA_{BD}$ -pSB3 C*	This study
pKan	*2μm, flp^- REP1 REP2 RAF kanMX* pTZ18R	(2)
pKanΔSTB	*2μm flp^- REP1 REP2 RAF STBΔ kanMX pTZ18R*	(11), pKanΔBg
pKanΔSTB-2μm-STB	pKanΔSTB *(2μm flp^- STBΔ)* + *2μm STB proximal*	(11), pKan-STB-P
pKanΔSTB-pSR1-Z	pKanΔSTB *(2μm flp^- STBΔ)* + *pSR1 Z*	This study
pKanΔSTB-pSB3-PAR	pKanΔSTB (*2μm flp^- STBΔ)* + *pSB3 PAR (pSB3 4989-5827)*	This study
pSB3-ARS-Kan	pSB3 *ARS* in pTZ18-Kan	This study
pSB3-ARS-Kan-pSR1-Z	pSB3-ARS-Kan + *pSR1 Z*	This study
pSB3-ARS-Kan-FLPp	pSB3-ARS-Kan + *pSB3 FLPp*	This study
pSB3-ARS-Kan-REP1p	pSB3-ARS-Kan + *pSB3 REP1p (839-bp, 4989-5827)*	This study
pSB3-ARS-Kan-REP1p-prox	pSB3-ARS-Kan + *pSB3 REP1p (*529-bp, pSB3 4989-5517)	This study
pSB3-ARS-Kan-REP1p-distal	pSB3-ARS-Kan + *pSB3 REP1p (*356-bp, pSB3 5472-5827)	This study
pSB3-ARS-Kan-pSB3-REP1p-581	pSB3-ARS-Kan + *pSB3 581-bp REP1p* (pSB3 5036-5616)	This study
pSB3-ARS-Kan-REP1-ORF	pSB3-ARS-Kan + *REP1* ORF nt 1132-1460	This study
pSB3-Kan	All pSB3 in pUC18-Kan: *FLP^+ $REP1^+$ C^+ kanMX*	This study
pSB3-flpΔ-Kan	pSB3-Kan *flpΔ*	This study
pSB3-REP1Δ123-Kan	pSB3-Kan *REP1(codons 1-254/378-517)*	This study
pSB3-flpΔREP1Δ123-Kan	pSB3-Kan *flpΔ, REP1(codons 1-254/378-517)*	This study
pTZ18R	*E. coli ori, Amp^r*	Pharmacia
pTZ18R-pSB3-B1	XhoI pSB3-B form cloned in SalI pTZ18R	This study
pTZ18R-pSB3-B2	XhoI pSB3-B form cloned in SalI pTZ18R	This study
pUC18::kanMX::ARS-pKW1/CEN	*kanMX, pKW1 ARS and L.waltii CEN in* pUC18	(3)
pUC18-Kan	*kanMX* in pUC18	This study

Table S3. Oligonucleotides used as PCR primers in the study

Name	Sequence (5'-to-3') *	Description
pKW1-REP1-F	cggaattcAAGATGAGTGAAGCTCAAA	Generate pKW1 *REP1* ORF with EcoRI/SalI overhangs
pKW1-REP1-R	cgagtcgACTATTATCCTTATTTCCTG	
pSR1-REP1-F	cagaattcAATATGTTTACTAGTCAGG	Generate pSR1 *REP1* ORF with EcoRI/SalI overhangs
pSR1-REP1-R	gagtcgACACGGTCTCAATAGTC	
pSR1-ORFS-F	caggatCCAGATGCAAATACAAAAC	Generate pSR1 *S* ORF with BamHI/SalI overhangs
pSR1-ORFS-R	gagtcgaCAATCCTAATCTATTAGCC	
pSB3-REP1-F	cagaattcGGTATGAACTTTGATCTTGATAG	Generate pSB3 *REP1* ORF with EcoRI/SalI overhangs
pSB3-REP1-R	gagtcgACTATACACTATGCGTCAG	
pSB3-ORFC-F	caggatccaaATGCCTCCTAGACGTAC	Generate pSB3 *C* ORF with BamHI/SalI overhangs
pSB3-ORFC-R	gagtcgacAACAAAGCAGTTTGACAAC	
pSB3-PAR-Bg	caGATCTATCAAGATCAAAGTTC	Amplify pSB3 839-bp *REP1* promoter (*REP1p*) with BglII/BamHI overhangs
pSB3- PAR -Bm	caggatCCACGCACAAACAAGTAC	
pSB3- PAR4-Bm	caggatcCGTTTGAGCTGCTTTCC	With pSB3-PAR6-Bg, amplify 455-bp pSB3 *REP1p (REP1* ORF proximal) with BglII/BamHI overhangs
pSB3- PAR6-Bg	caagatCTGTAGCACTCAAGCAAATG	Amplify pSB3 581-bp of *REP1p (REP1* ORF proximal) with BglII/BamHI overhangs
pSB3- PAR7-Bm	gaggatCCAATTGATGATGCGCTG	
pSB3- PAR8-Bg	caagatCTGGAAAGCAGCTCAAAC	With pSB3-PAR-Bm, amplify 356-bp pSB3 *REP1p (REP1* ORF distal) with BglII/BamHI overhangs
pSB3-FlpP-Bg	cagatctCATTTTAATTCGGTTATCTTG	Amplify pSB3 755-bp *FLP-C* promoter region with BglII and BamHI overhangs
pSB3-FlpP-Bm	caggatccCATACTTTTTATGTGTGATG	
pSB3-10	gagaaTTCATTCACTGTTCGAAATG	Amplify pSB3 *ARS* region as 383-bp SstI/EcoRI with BglIII site
pSB3-11	cagagctcagaTCTCCCACCGCCAGG	
pSR1-Z-Bm	caggatccGATGATTGTTTATGGCTCC	Amplify pSR1 *Z* locus as 523-bp with BamHI overhangs
pSR1-Z-Bm2	caggatccATAGTCGGTACCCGTAG	
pSB3-REP1-254-R	cttcggatCCGCTTTCATTTTCGCTTC	With pSB3-REP1-F generate pSB3 $REP1_{1\text{-}254}$ with EcoRI/BamHI overhangs
pSB3-REP1-378-F	gaaagcggatccGAAGCTGAAACTGCAGAAG	With pSB3-REP1-R generate pSB3 $REP1_{378\text{-}517}$ with BamHI/SalI overhangs
pSB3REP1-R143	cagtcgACTGCCGCGGAACGCC	With pSB3-REP1-F generate pSB3 $REP1_{1\text{-}143}$ with EcoRI/SalI overhangs
pSB3REP1-F70	gagaattcTCTAATGTACGTGGCATG	With pSB3-REP1-R generate pSB3 $REP1_{70\text{-}517}$ with EcoRI/SalI overhangs
pSB3-C60-R	cagtcgacTCTCTAGTAGGGAGATATTTC	With pSB3-ORFC-F generate pSB3 $C_{1\text{-}59}$ with BamHI/SalI overhangs
pSB3-C61-F	ctggatccgaaccggtGAGACTTTGCAGGAGATG	With pSB3-ORFC-R generate pSB3 $C_{61\text{-}213}$ with BamHI/SalI overhangs

* Lower case letters represent non-yeast plasmid linker sequences

Table S4. FoldSeek searches for structural similarities between 2μm family plasmid-encoded proteins (excluding Flp)

FoldSeek Searches			Accession numbers (truncated sequences in red)		Query Protein Size (aas)	Predicted Domains			Hits			truncated hit sequences in red		AFDB50 hits	AFDB-Swiss Prot hits
Query Protein	Plasmid	Species	Genbank	AFDB		1	2	3	Protein name	Plasmid	Species	AFDB accession #	Position in Query	E-value	E-value
Rep1	2μm	*S. cerevisiae*	P03871	P03871	373	2-108	131-183, 299-365	200-213, 222-291	Rep1	2μm	*S. uvarum*	A0AA35JL60	1-373	5.12E-44	
									Rep1	2μm	*S. cerevisiae x S. kudriavzevii*	H0H002	1-295	1.52E-28	
									Rep1	pKW1	*Lachancea sp. 'fantastica'*	A0A1G4K477	4-370	8.26E-22	
									Rep1 (B)	pSR1	*Z rouxii*	P13778	2-369	6.04E-20	1.47E-20
									Rep1	pTD1	*T. delbrueckii*	O43094	3-372	3.23E-16	
									Rep1	pSB2	*Z. bailii*	P13776	1-336	4.25E-15	1.04E-15
									Rep1 (B)	pSB3	*Z. rouxii*	A0A1Q3A2B5	1-368	2.55E-10	
									Rep1 (C)	pSM1	*Z. fermentatii*	P13742	181-367	1.67E-08	4.07E-09
									Rep1 (D)	pKW1	*L. waltii*	Q01090	1-278	1.59E-07	
									Rep1 (B)	pSB3	*Z. rouxii*	P13777	214-372	2.09E-06	5.09E-07
Rep1	pTD1	*T. delbrueckii*	CAA71932.1	O43094	397	37-142	177-378	0	Rep1 (B)	pSR1	*Z rouxii*	P13778	32-397	5.46E-19	
									Rep1	pKW1	*Lachancea sp. 'fantastica'*	A0A1G4K477	38-380	1.47E-17	
									Rep1	pSB2	*Z. bailii*	P13776	32-343	2.24E-13	
									Rep1	2μm	*S.uvarum*	A0AA35JL60	35-383	7.41E-12	
									Rep1	pKD1	*K. lactis*	P13775	6-393	8.45E-11	
									Rep1 (D)	pKW1	*L. waltii*	Q01090	16-309	9.70E-08	
									Rep1 (C)	pSM1	*Z. fermentatii*	P13742	212-381	1.32E-07	
									Rep1 (B)	pSB3	*Z. rouxii*	A0A1Q3A2B5	36-189	1.25E-07	
									Rep1 (B)	pSB3	*Z. rouxii*	P13777	144-384	4.25E-05	
									Rep1	2μm	*S. cerevisiae x S. kudriavzevii*	H0H002	24-330	2.05E-04	
Rep2	2μm	*S. cerevisiae*	P03872	P03872	296	1-61, 84-107	123-233	0	no hits						
C	pTD1	*T. delbrueckii*	CAA71933.1	O43095	240	68-100	112-181	0	no hits						
C	pKW1	*L. waltii*	CAA39901	Q01086	306	135-165	167-258	0	C	pKW1	*L. mirantina*	A0A1G4JFN4	1-257		5.48E-10
									A	pSM1	*L. fermentati*	P13740	94-257		2.57E-05
C	pKW1	*L. mirantina*	SCU89113 (gene	A0A1G4JFN4	308	100-160	170-291	0	C	pKW1	*L. waltii*	Q01086	1-308	4.85E-10	
									A	pSM1	*L. fermentati*	P13740	89-308	9.78E-06	
A	pSM1	*L. fermentati*	P13740	P13740	260	14-112	121-215	0	C	pKW1	*L. waltii*	Q01086	5-260	1.20E-05	
									C	pKW1	*L. mirantina*	A0A1G4JFN4	33-259	2.79E-05	
									Rep2	2μm	*S. cerevisiae*	P03872	2-260		9.10E-04
C	pSB2	*Z. bailii*	P13780	P13780	249	no domains identified			S	pSR1	*Z. rouxii*	P13782	1-233	1.45E-05	
S	pSR1	*Z. rouxii*	P13782	P13782	233	2-60	84-190	0	C	pSB2	*Z. bailii*	P13780	21-212	5.85E-05	1.43E-05
C	pSB3	*Z. rouxii*	ASW33869	A0A286RWR0	213	13-72	93-174	0	no hits						
C	pKD1	*K. lactis*	P13779	P13779	212	2-50	0	0	no hits						
Raf	2μm	*S. cerevisiae*	AAB59342.1	Q06891	181	5-71	81-152	0	no hits						
B	pKW1	*L. waltii*	CAA39903	Q01110	241	27-89, 104-132	133-216	0	no hits						
B	pSM1	*L. fermentati*	P13741	P13741	224	25-125	128-209	0	no hits						

Table S5. Other 2μm family Rep1 protein sequences analyzed

Protein Name	Plasmid	Species	Accession numbers (truncated sequences in red)		Protein Size (aas)	Predicted Domains		
			Genbank #	AFDB #		1	2	3
Rep1	pSB2	*Z. bailii*	P13776 (missing C-terminal 82 aas)	P13776				
Rep1 (P)	pSR1	*Z. rouxii*	P13778	P13778	410	5-119	138-223, 265-393	0
Rep1 (B)	pSB3	*Z. rouxii*	P13777 (missing N-terminal 195 aas)	P13777				
Rep1	pSB3	*Z. rouxii*	XPQ57843.1		517	Domain analysis not available		
Rep1	pSB3	*Z. rouxii*	ZYGR_0R01040 (gene integrated in genome)	A0A1Q3A2B5	460	10-134	152-205, 398-451	331-392
Rep1	pKW1	*Lachancea sp. 'fantastica'*	SCU98552 (gene integrated in genome)	A0A1G4K477	393	2-120	0	0
Rep1 (D)	pKW1	*L. waltii*	CAA39902 (missing C-terminal 126 aas)	Q01090				
Rep1 (D)	pKW1	*L. waltii*	XPQ57840.1		381	Domain analysis not available		
Rep1 (C)	pSM1	*L. fermentati*	P13742 (missing N-terminal 194 aas)	P13742				
Rep1	2μm	*S. uvarum*	CAI4065199 (gene integrated in genome)	A0AA35JL60	357	Domain analysis not available		
Rep1	2μm	*S. cerevisiae x S. kudriavzevii*	EHN00613.1 (gene integrated in genome)	H0H002	330	16-121	143-196	0

Supplementary Methods

kanMX-tagged pSB3-based plasmids

A *kanMX*-tagged version of pSB3 that could be propagated in yeast and *E.coli* (pSB3-Kan) was created by inserting a 3.2 kbp EcoRI-MluI fragment containing the origin of replication and ampicillin-resistance gene region from pUC18 and the *kanMX* cassette from pUG6 (12) between two MluI sites downstream of the pSB3 *C* gene. Other than the 9-bp MluI fragment replaced by vector insertion, and 24-bp of pTZ18R polylinker sequence retained at the original XhoI cloning site, all other pSB3 plasmid sequences are present and undisrupted in pSB3-Kan. To create a version of pSB3-Kan lacking a functional *FLP* gene (pSB3-flpΔ-Kan), pSB3-Kan was digested with AgeI and StuI, the overhangs filled in, and the plasmid circularized by ligation. Versions of pSB3-Kan with a *REP1* ORF lacking 123 codons (codons 255 to 377), either with an intact *FLP* ORF (pSB3-REP1Δ123-Kan), or with the *FLP* ORF deleted (pSB3-flpΔ-REP1Δ123-Kan), were created by replacement of a 1.0 kbp SstII/BglII fragment of pSB3-Kan and pSB3-flpΔ-Kan, respectively, with a 0.7 kbp SstII/BglII fragment isolated from a PCR-generated internally-deleted version of the *REP1* gene.

To create the *kanMX*-tagged yeast-*E.coli* shuttle vector (pSB3-ARS-Kan) that could replicate but not partition or be targeted by Flp recombinase in yeast, a 383-bp stretch of pSB3, containing the region previously shown to be required for autonomous replication in *S.cervisiae (4)*, and the adjacent *REP1* gene transcriptional termination sequence was amplified as a SstI/EcoRI fragment with a unique BglII cloning site and cloned in SstI/EcoRI-digested pTZ18R. Regions of pSB3 tested for partitioning function in Z. rouxii were amplified by PCR with flanking BglII and BamHI sites, creating the pSB3-ARS-Kan series of plasmids (see Tables S2 and S3).

Reporter Plasmids for One-Hybrid Protein-DNA Interaction Assays

Various regions from pSB3 were amplified from the native plasmid by PCR with primers that introduced flanking BglII and BamHI restriction sites (See Table S3). The resulting fragments were cloned at the BglII site in the *URA3* gene-based one-hybrid vector pJL638 upstream of the *lacZ* reporter gene, creating the pJL638 plasmid series, pSB3-REP1p, pJL638-pSB3-REP1p-581, pJL638-pSB3-REP1p-distal, and pJL638-pSB3-FLPp, respectively (Table S2). To test the proximal portion of the pSB3 *REP1* promoter for pSB3 partitioning protein recognition, pJL638-pSB3-REP1p was digested with SmaI and SpeI to remove 310-bp most distal from the *REP1* ORF, overhangs filled-in and the plasmid circularized, creating pJL638-pSB3-REP1p-prox. To integrate the reporter genes into the yeast genome, pJL638 plasmids were linearized with StuI, which cuts within the *URA3* gene in the pJL638 sequence and used to transform *S. cerevisiae* strain JP49/6b (lacks 2µm; *ura3-1*) to uracil prototrophy.

2µm-Based Plasmids

An amplification-defective *(FLP* gene disrupted*),* partitioning-competent, *kanMX*-tagged, 2µm-based plasmid (pKan), and pKan lacking the *STB* locus repeat region (pKanΔBg) (called pKanΔSTB here), have been previously described (2,11). To create a pKan-based plasmid that had the pSR1 partitioning sequence in place of the native 2µm *STB* repeats, the previously identified pSR1 partitioning locus (*Z* locus) (13) was amplified from the native plasmid by PCR with primers that introduced flanking BglII and BamHI restriction sites (See Table S3), and cloned at the BglII site in the plasmid pKanΔBg, generating plasmid pKanΔSTB-pSR1-Z. Similarly, the pSB3 *PAR* sequence used in the one-hybrid reporter plasmid was cloned at the BglII site in the plasmid pKanΔBg, creating pKanΔSTB-pSB3-PAR. Creation of pKanΔSTB with the 2µm STB repeat region re-introduced as a BglII fragment (pKanΔSTB-2µm-STB), has been previously described (11)

Plasmid copy number estimation

S. cerevisiae yeast cells lacking native 2µm (*cir*^{0}) were cultured to stationary phase under selective conditions (YPAD+G418) if transformed with a *kanMX*-tagged plasmid or if untransformed, were cultured in YPAD. Total genomic DNA was extracted as previously described (14), digested with EcoRI, resolved by agarose gel electrophoresis, visualized by ethidium bromide staining and imaged on a VersaDoc MP4000 (Biorad). Fluorescence intensity of the 3.4-kbp EcoRI fragment common to each pSB3-Kan-based plasmid relative to the 2.85-kbp host ribosomal DNA repeat fragment (copy number ~100-200 per haploid genome) (15) was determined by densitometry using ImageLab software.

Statistical analysis
For plating assays and plasmid copy number measurements, results represent the average with standard deviation from analyzing a minimum of four yeast transformants for each unless otherwise indicated. Statistical significance was assessed using a two-tailed Student's t-test.

Western blotting
Protein was extracted from yeast cultures and analyzed by western blotting as previously described (9). Rabbit polyclonal anti-LexA (Invitrogen), rabbit polyclonal anti-Gal4$_{AD}$ (Sigma) and mouse monoclonal anti-Pgk1 (Abcam) were used as primary antibodies with Horseradish peroxidase (HRP)-conjugated goat anti-rabbit (Novex) and anti-mouse IgG (Sigma) as secondary antibodies. Signals were detected by chemiluminescence using a Clarity Western ECL Substrate Kit (BioRad) and images captured using a VersaDoc 4000MP imaging system and Quantity One software (BioRad) or by autoradiography. Images were processed using Adobe Photoshop CS6 and figures prepared using Adobe Illustrator CS6.

Motif Searches
Predicted sequences of Rep1 proteins encoded by representative members of the 2μm family were searched for shared motifs using GLAM2 (Gapped Local Alignment of Motifs) (16).

Protein Models
Predicted three-dimensional structures of partitioning proteins encoded by members of the 2μm family of plasmids were obtained by searching the AlphaFold Protein Database (AFDB at EMBL-EBI (alphafold.ebi.ac.uk) (17,18) or generated using AlphaFold (alphafoldserver) (17). Predicted domains were obtained from The Encyclopedia of Domains (19) accessed through AFDB. Similarities between predicted protein structures were predicted using Foldseek (20), accessed through AFDB or the Foldseek server (search.foldseek). Images were processed using Adobe Photoshop CS6 and figures prepared using Adobe Illustrator CS6.

Supplementary References

1. Dobson, M.J., Pickett, A.J., Velmurugan, S., Pinder, J.B., Barrett, L.A., Jayaram, M. and Chew, J.S.K. (2005) The 2μm plasmid causes cell death in yeast with a mutation in Ulp1 protease. *Mol.Cell. Biol.*, **25**, 4299-4310.
2. Pinder, J.B., McQuaid, M.E. and Dobson, M.J. (2013) Deficient sumoylation of yeast 2-micron plasmid proteins Rep1 and Rep2 associated with their loss from the plasmid-partitioning locus and impaired plasmid inheritance. *PLoS One*, **8**, e60384.
3. Di Rienzi, S.C., Lindstrom, K.C., Lancaster, R., Rolczynski, L., Raghuraman, M.K. and Brewer, B.J. (2011) Genetic, genomic, and molecular tools for studying the protoploid yeast, *L. waltii*. *Yeast*, **28**, 137-151.
4. Toh-e, A. and Utatsu, I. (1985) Physical and functional structure of a yeast plasmid, pSB3, isolated from *Zygosaccharomyces bisporus*. *Nucleic Acids Res*, **13**, 4267-4283.
5. Ortiz-Merino, R.A., Kuanyshev, N., Braun-Galleani, S., Byrne, K.P., Porro, D., Branduardi, P. and Wolfe, K.H. (2017) Evolutionary restoration of fertility in an interspecies hybrid yeast, by whole-genome duplication after a failed mating-type switch. *PLoS Biol*, **15**, e2002128.
6. Toh-e, A., Araki, H., Utatsu, I. and Oshima, Y. (1984) Plasmids resembling 2μm DNA in the osmotolerant yeasts *Saccharomyces bailii* and *Saccharomyces bisporus*. *J.Gen.Microbiol.*, **130**, 2527-2534.
7. Li, J.J. and Herskowitz, I. (1993) Isolation of ORC6, a component of the yeast origin recognition complex by a one-hybrid system. *Science*, **262**, 1870-1874.
8. Sengupta, A., Blomqvist, K., Pickett, A., Zhang, Y., Chew, J.S.K. and Dobson, M.J. (2001) Functional domains of yeast plasmid-encoded Rep proteins. *J.Bacteriol.*, **183**, 2306-2315.
9. McQuaid, M.E., Pinder, J.B., Arumuggam, N., Lacoste, J.S.C., Chew, J.S.K. and Dobson, M.J. (2017) The yeast 2-micron plasmid Raf protein contributes to plasmid inheritance by stabilizing the Rep1 and Rep2 partitioning proteins. *Nucl. Acids Res.*, **45**, 10518-10533.

10. Lech, K., Anderson, K. and Brent, R. (1988) DNA-bound Fos proteins activate transcription in yeast. *Cell*, **52**, 179-184.
11. McQuaid, M.E., Polvi, E.J. and Dobson, M.J. (2019) DNA sequence elements required for partitioning competence of the *Saccharomyces cerevisiae* 2-micron plasmid *STB* locus *Nucl. Acids Res.*, **47**, 716-728.
12. Guldener, U., Heck, S., Fielder, T., Beinhauer, J. and Hegemann, J.H. (1996) A new efficient gene disruption cassette for repeated use in budding yeast. *Nucleic Acids Res*, **24**, 2519-2524.
13. Araki, H., Awane, K., Irie, K., Kaisho, Y., Naito, A. and Oshima, Y. (1993) A specific host factor binds at a cis-acting transcriptionally silent locus required for stability control of yeast plasmid pSR1. *Mol Gen Genet*, **238**, 120-128.
14. Dobson, M.J., Yull, F.E., Molina, M., Kingsman, S.M. and Kingsman, A.J. (1988) Reconstruction of the yeast 2µm plasmid partitioning mechanism. *Nucl.Acids Res.*, **16**, 7103-7117.
15. Kobayashi, T., Heck, D.J., Nomura, M. and Horiuchi, T. (1998) Expansion and contraction of ribosomal DNA repeats in *Saccharomyces cerevisiae*: requirement of replication fork blocking (Fob1) protein and the role of RNA polymerase I. *Genes Dev*, **12**, 3821-3830.
16. Frith, M.C., Saunders, N.F., Kobe, B. and Bailey, T.L. (2008) Discovering sequence motifs with arbitrary insertions and deletions. *PLoS Comput Biol*, **4**, e1000071.
17. Jumper, J., Evans, R., Pritzel, A., Green, T., Figurnov, M., Ronneberger, O., Tunyasuvunakool, K., Bates, R., Zidek, A., Potapenko, A. *et al.* (2021) Highly accurate protein structure prediction with AlphaFold. *Nature*, **596**, 583-589.
18. Fleming, J., Magana, P., Nair, S., Tsenkov, M., Bertoni, D., Pidruchna, I., Lima Afonso, M.Q., Midlik, A., Paramval, U., Zidek, A. *et al.* (2025) AlphaFold Protein Structure Database and 3D-Beacons: New Data and Capabilities. *J Mol Biol*, **437**, 168967.
19. Lau, A.M., Bordin, N., Kandathil, S.M., Sillitoe, I., Waman, V.P., Wells, J., Orengo, C.A. and Jones, D.T. (2024) Exploring structural diversity across the protein universe with The Encyclopedia of Domains. *Science*, **386**, eadq4946.
20. van Kempen, M., Kim, S.S., Tumescheit, C., Mirdita, M., Lee, J., Gilchrist, C.L.M., Soding, J. and Steinegger, M. (2024) Fast and accurate protein structure search with Foldseek. *Nat Biotechnol*, **42**, 243-246.

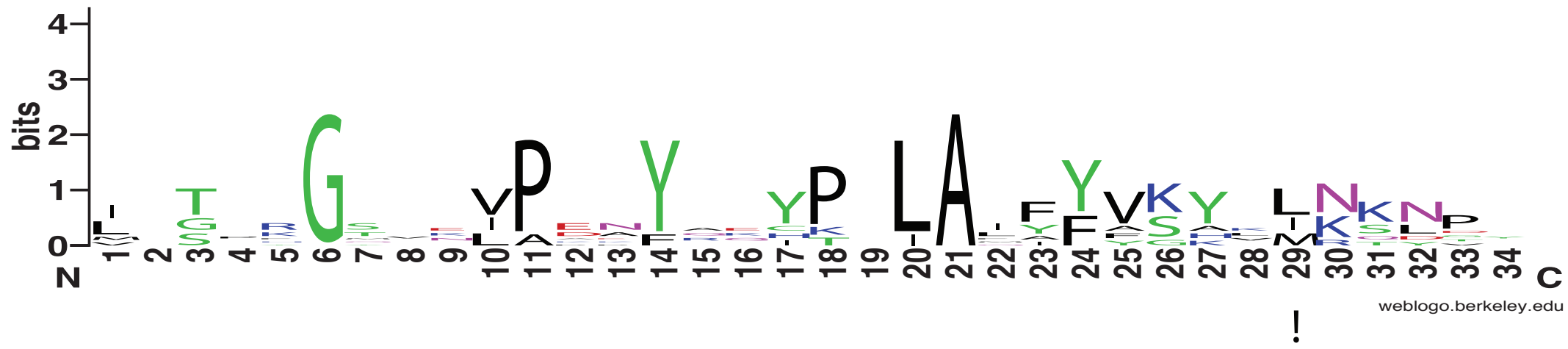


```
Motif:[IL][VI][TGS]P?[RK]G[TS]V[NEK][VIL]P[DE][NA]Y[ARQ][KEQ]YP[IQ]LA[IL][YF][FY]V[KS]
Y[VLK][LMI][NK][KS][LN]PY Score: 257.878
                            **********************************
2um             30 IETTRGTFPVPDNYKKYKTLAFAFVGHVLNTDDT  63 + 29.3
pTD1            62 VVGMRGYMNVPDPYAEYPMLAIYFAKYRLRKYPF  95 + 43.3
pSR1            39 LSTAKGMVNIAENYRDYPILAIFYVKYLMKKLPY  72 + 60.5
pKD1            56 IITQHGSYEVPEAFAQCTYLAQIYVSYKINSLPY  89 + 35.2
pKW1            40 LVSPYGSTKLPKEYSEHPQLALFYYSYMINKNTA  73 + 42.8
pSB2            39 LITPKGTVEIPENYQNYPILAIFYVKYLMKKNPY  72 + 74.5
pSM1            40 MQSPFGSVSLPAKYQQYPQLALFYFSAVLNQNGL  73 + 39.1
pSB3            41 ILG.RGQAKVPPAYRKIPLIAMYFVKKKLKSNVR  73 + 25.9
```

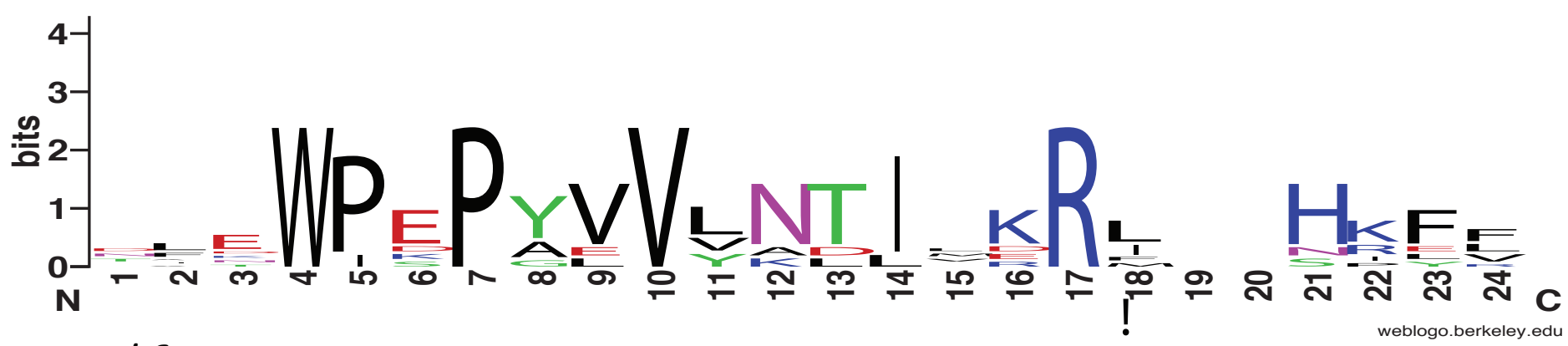


```
Motif: [DNT][LF]EWPEP[AY]VV[YLV]NTI[VML]KR[IL][QE]KH[RK]F[LFV] Score: 280.535
                            ************************
2um             69 ELDWPDPALVYNTIVDRIINHPEL  92 + 30.3
pTD1           101 DITWPEPYVVLNTIMRRLRTHRFL 124 + 56.2
pSR1            78 NLEWPEPYVVLNTILKRLKEHKFF 101 + 65.9
pKD1            95 DLEWISPAEVYKLIMERLQKNKYF 118 + 23.9
pKW1            81 TFEWPKPYVVLNTIVKRLESHIFV 104 + 55.8
pSB2            78 TVNWPEPYVVVNTITKRFQDHKLF 101 + 48.8
pSM1            81 NFEWPEPYVVLNTLIKRIEQHRFV 104 + 58.6
pSB3            79 AAKWPEPGVVVADILKRMNKSKFR 102 + 21.0
```

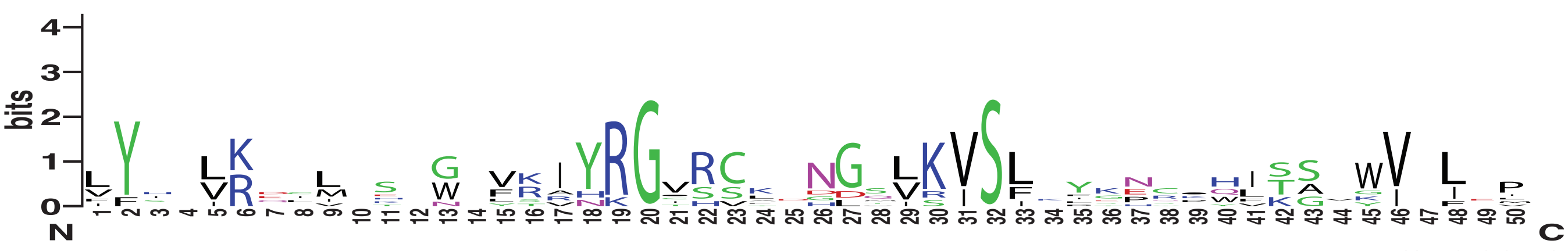


```
Motif:[LV]Y[HSI]I[LV][KR][EDQ][LCI][LM]GS[KS][GW][KE][VF][KR]IYRGV?[SR][SC][LK]DNG[SQ]
[VL]IV]S[LF]KY[KG][ENP][RC][KP]?[HQW][IL][TKS][ASG]VW[VI][SN][LI]EP  Score: 370.138
                            **************************************************
2um            300 LYHILKELIQSQGTVKVYRG.SSFSHDSIKISLHYEEQ.HITAVWVYLTV 347 + 54.1
pTD1           307 IYSKIRAWLPKKNKVKIYRGTSVLEDGSVKVSLNFTEYSYIKSAYISFRP 356 + 44.3
pSR1           318 LYLDLKGCLGSSWKFSIYRGVRCKQNGQLKVSLKPSNSGHLSGFWVNIKM 367 + 77.1
pKD1           332 LFHTVRDVLLHDGSYTRHKGVRCLDNGSVRVSIRLKHR.QLTATWIELEP 380 + 48.3
pKW1           304 VYSFVRQIMFSEGRVKINRGVHCTDNGMVKISLSYGPCKWIKSVGVSLAI 353 + 72.8
pSB2           324 FYINLKECLGTSWEFRIYRGIRCKKNGQLKVSFKIKNCPHISSYWVNIEL 373 + 94.3
pSM1           313 VYGYVRQIMYSKGQVRIYRGVRCKPNGVLKVSLFYGPCKWVTSMWVTLVP 362 + 87.6
pSB3           440 LYIILKDLVEEPWELRAYRGCRSADGLTLSVSFKYKNRPQFSGVKVDLEP 489 + 51.2
```

Figure S1. Shared Rep1 sequence motifs. Rep1 sequences encoded by the indicated plasmids were searched for shared motifs using GLAM2 (16). Scores for each match are shown at right, with the start and end positions of the matched part of each sequence indicated. The logo above (generated with Weblogo (http://weblogo.berkeley.edu), represents the consensus.

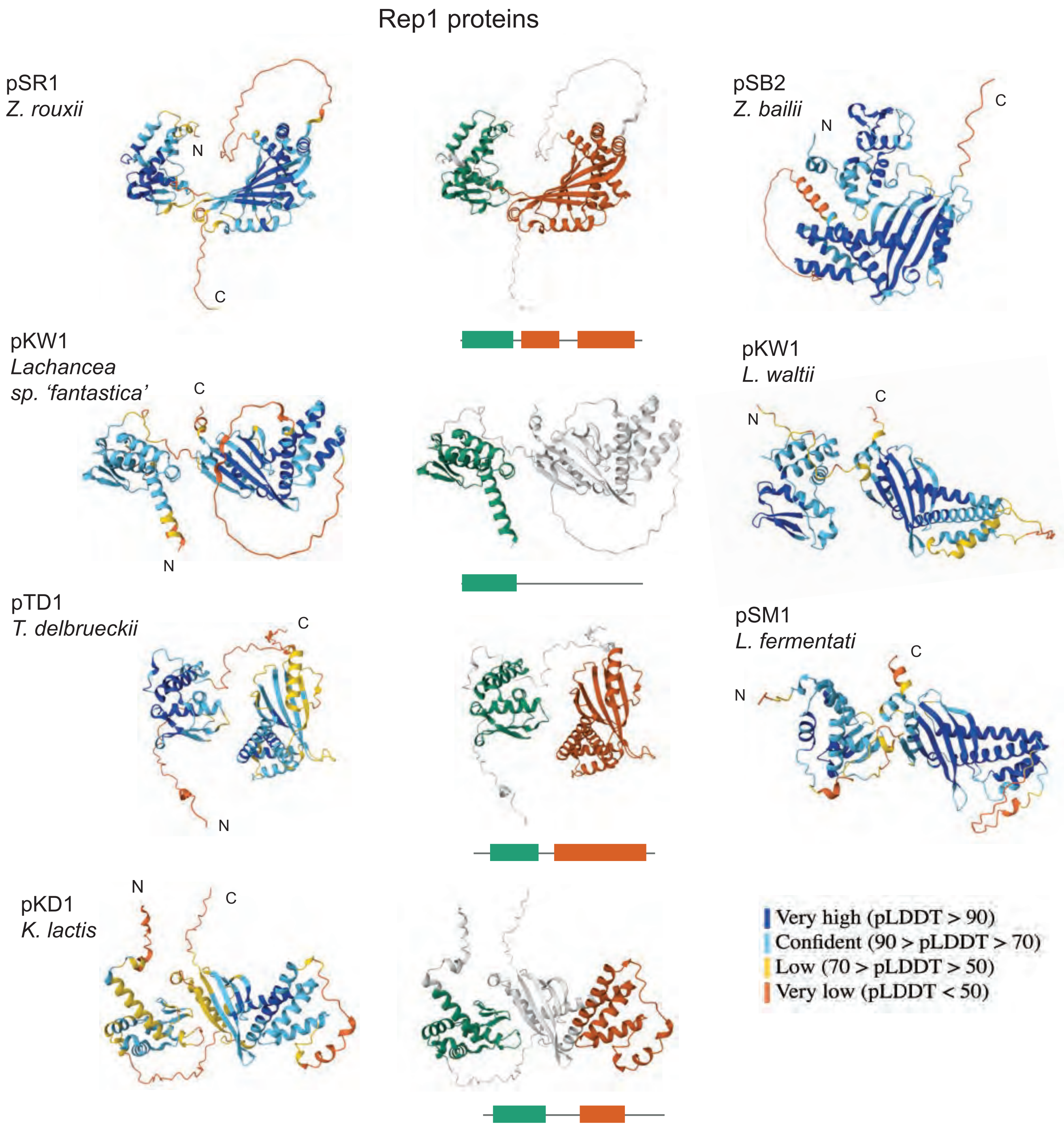

Figure S2. Predicted structures of Rep1 proteins encoded by other 2μm family plasmids.
Predicted structures and domains of Rep1 proteins encoded by pSR1 (P13778), pKW1 (A0A1G4K477), pTD1 (O43094), and pKD1 (P13775) were obtained from the Alphafold Protein Database and The Encyclopedia of Domains. Domain predictions are shown at right with domain positions in sequence displayed schematically beneath. Models for Rep1 proteins encoded by pKW1 (XPQ57840), pSB2 (CP019507.2) and pSM1 (M18275 with C nucleotide added after nucleotide 2804) were generated using AlphaFold. Amino (N) and carboxy (C) termini are indicated. Structures are shown with colours indicating the per-residue model confidence score (pLDDT) between 0 and 100.

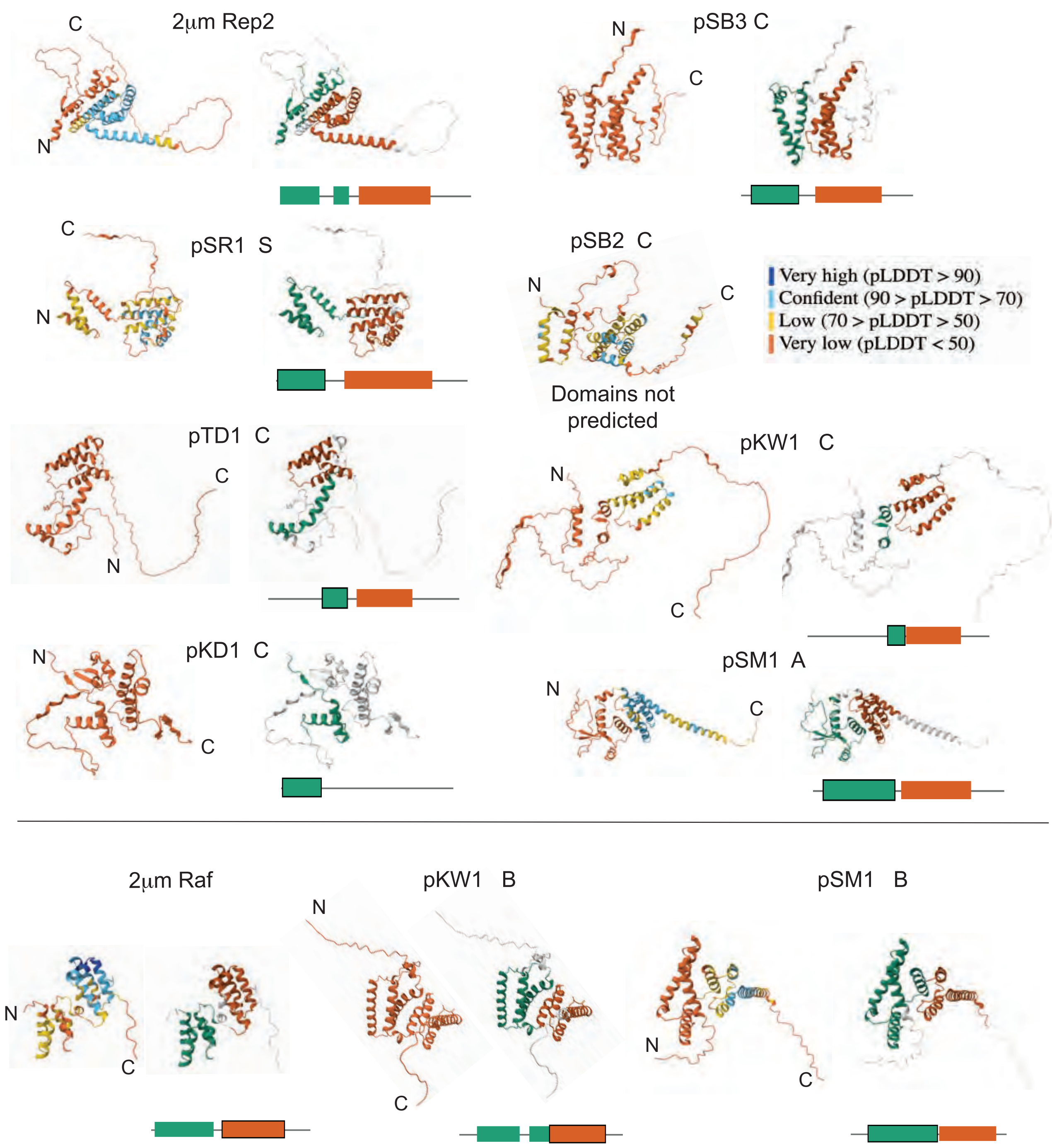

Figure S3. Predicted structures of proteins encoded by the 2µm *REP2* and *RAF* genes and those encoded by similarly-positioned ORFs in other members of the 2µm family of plasmids. AlphaFold models of *S. cerevisiae* 2µm Rep2 (P03872) and Raf (Q06891), *Z. rouxii* pSR1 protein S (P13782), *T. delbrueckii* pTD1 protein C (O43095), *K. lactis* pKD1 protein C (P13779), pSB3 protein C (A0A286RWR0), *Z. bailii* pSB2 protein C (P13780), *L. waltii* pKW1 protein protein C (Q01086) and protein B (Q01110) and *L. fermentati* pSM1 protein A (P13740) and protein B (AP13741) are shown coloured according to the per-residue model confidence score (pLDDT) (*left*). Where predicted, domains are shown indicated by colour (*right*) with position of sequences comprising each domain shown schematically beneath. Amino (N) and (C) carboxy termini are indicated. Models and domain predictions were obtained from the Alphafold Protein Database and The Encyclopedia of Domains.

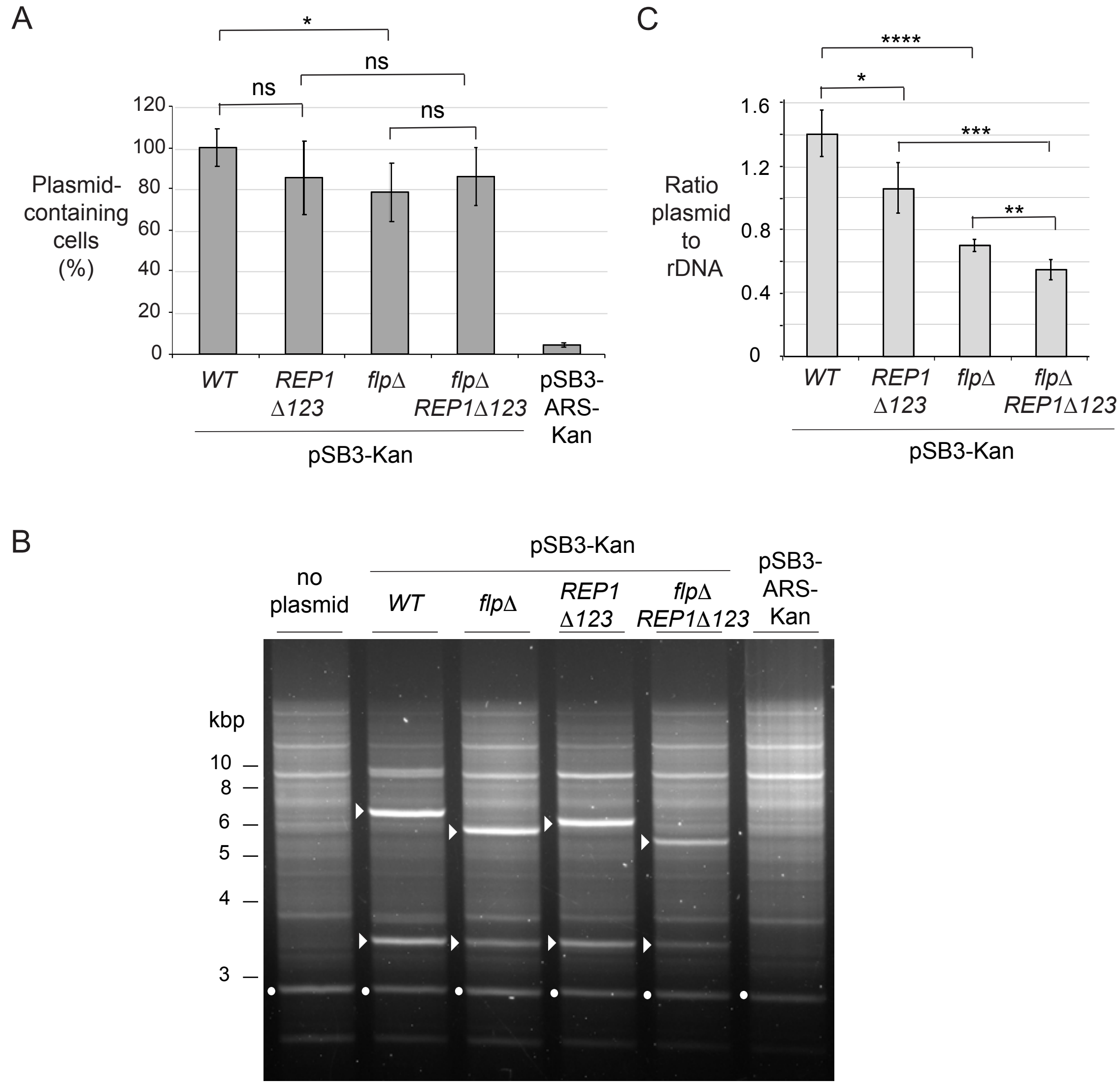

Figure S4. Effect of deletion of the pSB3-Kan *FLP* gene or *REP1* codons 255-377 on plasmid mitotic stability and copy number. *S. cerevisiae* cells lacking 2μm plasmid were transformed with pSB3-Kan with all three pSB3 protein-coding genes intact (WT), or with the *FLP* gene (*flp*Δ), or *REP1* codons 255-377 (*REP1Δ123*), deleted as indicated. Transformants were cultured in selective medium (YPAD containing G418) and if untransformed, in YPAD. (**A**) The percentage of cells containing plasmid for each transformant was determined by a plating assay. (**B**) Genomic DNA was extracted, digested with EcoRI and resolved by agarose gel electrophoresis. A representative gel is shown with EcoRI fragments of the sizes expected for plasmid products (arrowheads) and the host strain 2.85-kbp ribosomal DNA fragment (white circles) indicated. (**C**) Fluorescence intensity of the 3.4-kbp EcoRI fragment common to each pSB3-Kan-based plasmid relative to the 2.85-kbp host rDNA repeat fragment (copy number ~100 per genome) was determined by densitometry. Note that pSB3-ARS-Kan copy number is too low for the plasmid 4.7-kbp EcoRI fragment to be seen above the background of genomic DNA fragments. Results represent the average (±StDev) (n=4) (ns= not significant, *p<0.05, **p<0.01, ***p<0.001, ****p<0.0001)

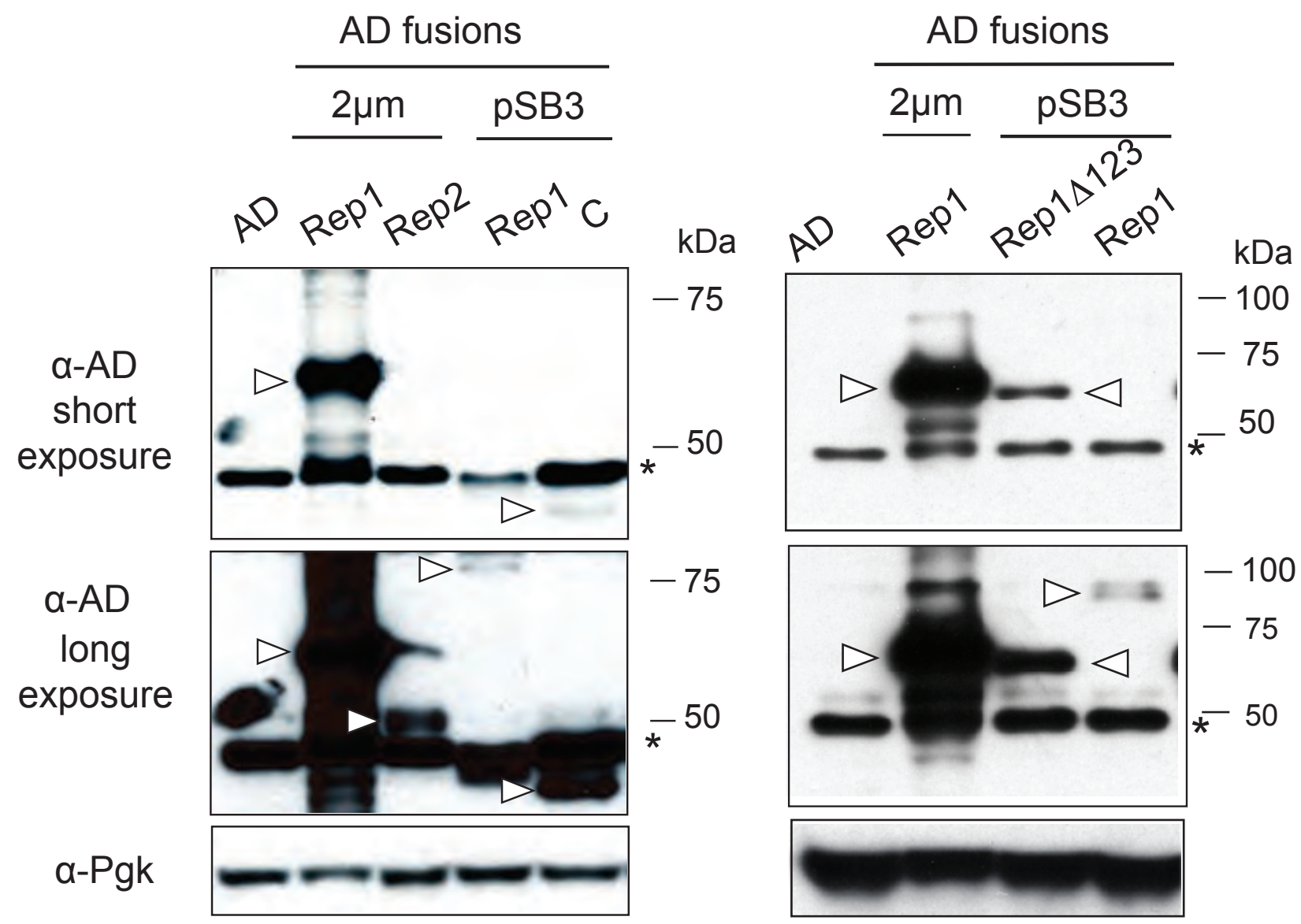

Figure S5. Steady-state levels of pSB3 and 2μm partitioning proteins expressed in *[cir⁰] S. cerevisiae* yeast in the absence of their partner partitioning protein. Total protein was extracted from yeast transformants expressing $Gal4_{AD}$ (AD) or the indicated 2μm or pSB3 protein fused to AD. Proteins were resolved by SDS-PAGE and analyzed by western blotting with antibodies specific for AD (α-AD), or Pgk (α-Pgk). Species with the mobility expected for each of the protein fusions are indicated with arrowheads. A non-target yeast protein detected by the α-AD antibody is indicated with an asterisk.

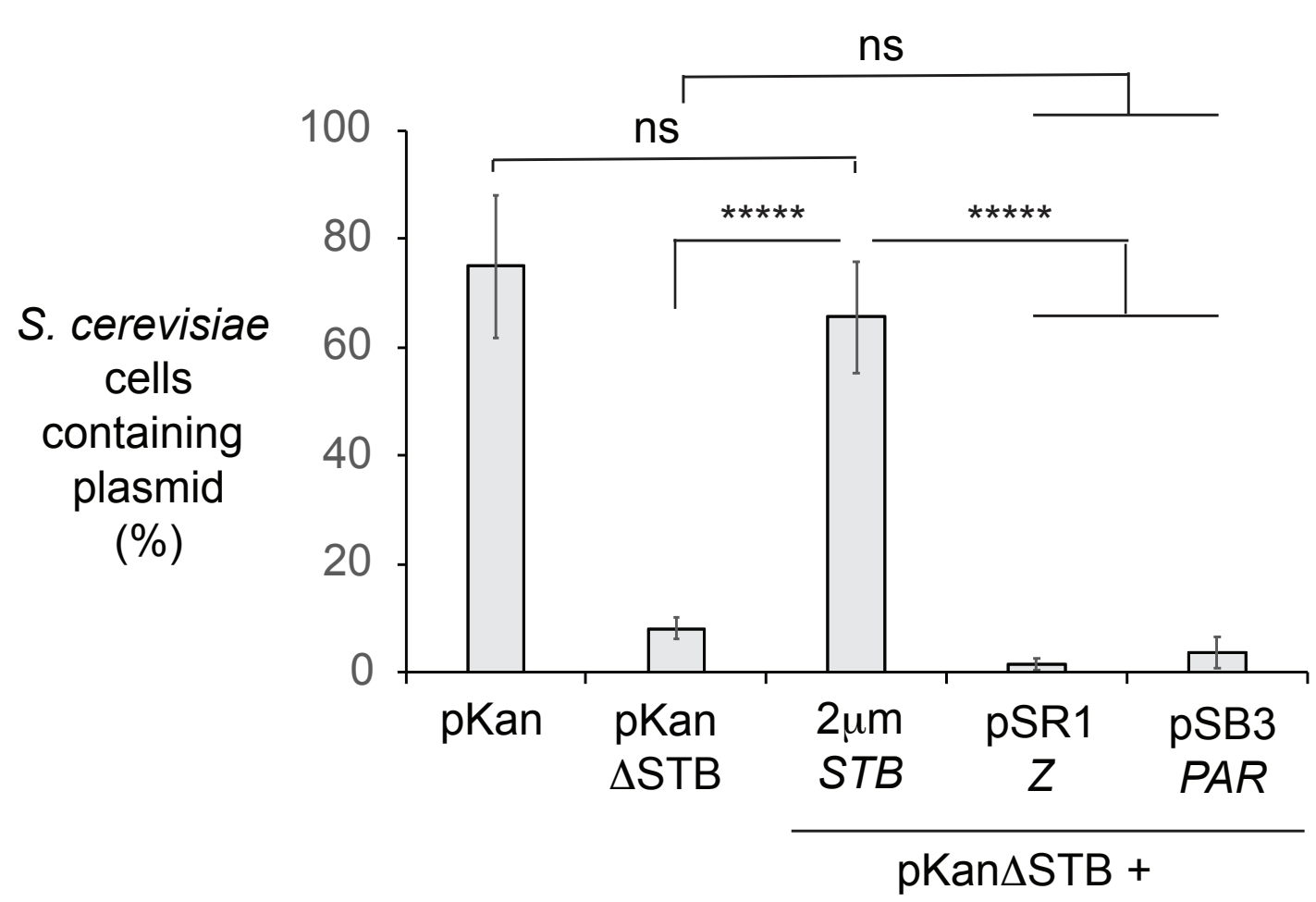

Figure S6. The pSR1 and pSB3 partitioning loci cannot functionally substitute for 2μm *STB*. *S. cerevisiae* cells lacking 2μm were transformed with *kanMX*-tagged plasmids: an amplification-defective 2μm-based plasmid encoding *REP1*, *REP2* and the native *STB* partitioning locus (pKan), or pKan from which the *STB-proximal* repeat region had been deleted (pKanΔSTB), or deleted and replaced with 2μm *STB-proximal*, or the pSR1 *Z* sequence, or the pSB3 *PAR* sequence (pSB3 nt 4989-5827). Transformants were cultured in selective medium (YPAD containing G418) for ~10 generations and the percentage of cells resistant to G418 (an indication of presence of a *kanMX*-tagged plasmid) was determined by a plating assay. Results represent the average (± StDev) from assaying six independent transformants for each plasmid. Statistical significance of difference was calculated using a two-tailed Student's t-test (ns = not significant; ***** $p<0.000001$).
